# “Rate-limited effect” of reverse intersystem crossing process: the key for tuning thermally activated delayed fluorescence lifetime and efficiency roll-off of organic light emitting diodes†

**DOI:** 10.1039/c6sc00542j

**Published:** 2016-03-15

**Authors:** Xinyi Cai, Xianglong Li, Gaozhan Xie, Zuozheng He, Kuo Gao, Kunkun Liu, Dongcheng Chen, Yong Cao, Shi-Jian Su

**Affiliations:** a State Key Laboratory of Luminescent Materials and Devices and Institute of Polymer Optoelectronic Materials and Devices, South China University of Technology Guangzhou 510640 China mssjsu@scut.edu.cn

## Abstract

Issues concerning excited state lifetime (*τ*_TADF_) tuning of thermally activated delayed fluorescence (TADF) materials are critical for organic light emitting diode (OLED) applications and other specific fields. For TADF-OLEDs, employing emitters with a short *τ*_TADF_ gives rise to suppressed singlet–triplet annihilation (STA) and triplet–triplet annihilation (TTA), leading to reduced efficiency roll-off at practical relevant brightness (100 and 1000 cd m^−2^ for display and illumination applications, respectively). Through molecular design, exciton dynamic process rate constants including fluorescence (*k*_F_), intersystem crossing (*k*_ISC_), internal conversion (*k*_IC_) and reverse intersystem crossing (*k*_RISC_) are selectively altered, affording four representative TADF emitters. Based on lifetime and quantum yield measurements, *k*_F_, *k*_ISC_, *k*_IC_ and *k*_RISC_ are calculated for four emitters and their interrelationship matches corrected time-dependent density functional theory simulation. Among them, even with a small *k*_F_, low photoluminescence quantum efficiency (*Φ*) and large *k*_ISC_, molecules with a small singlet–triplet splitting energy (Δ*E*_ST_) and lowest charge transfer triplet excited state (^3^CT) eventuate in shortening the *τ*_TADF_. Herein, *k*_RISC_, which is inversely proportional to Δ*E*_ST_, turns out to be the rate-limited factor in tuning the *τ*_TADF_ (“rate limited effect” of the RISC process). As revealed by flexible potential surface scanning, PyCN–ACR exhibited a moderate *k*_F_, reduced *k*_IC_ and enlarged *k*_RISC_, resulting in a short *τ*_TADF_ and a moderate *Φ* with orange-red emission. OLEDs containing PyCN–ACR as the emitting guest achieved orange-red TADF-OLEDs with an emission peak at 590 nm and the best external quantum efficiencies (EQEs) of 12.4%/9.9%/5.1% at practical luminances of 100/1000/10 000 cd m^−2^.

## Abstract 2

The rate constant of reverse intersystem crossing was found to be the “rate-limited step” in thermally activated delayed fluorescence lifetime governing.

## Graphical abstract

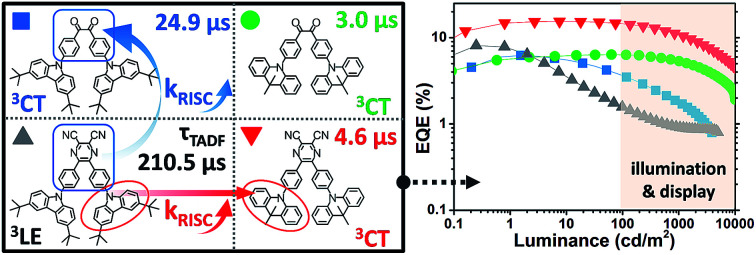

## Introduction

**Scheme 1:**
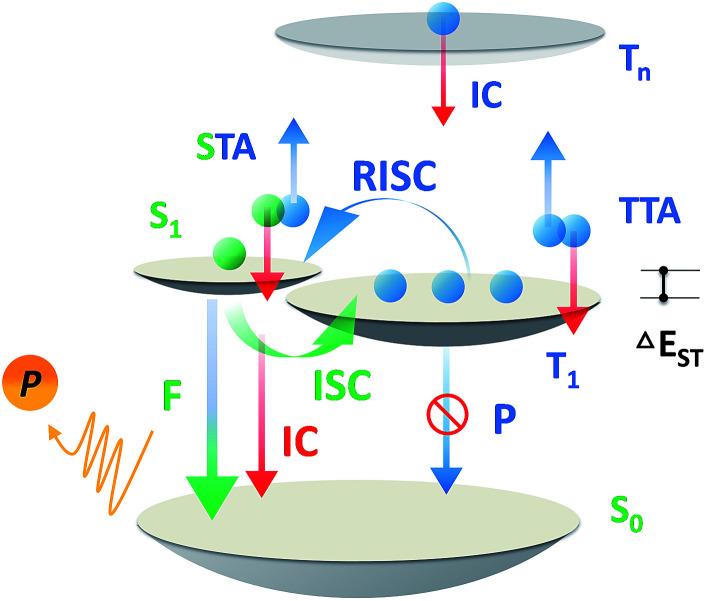
Exciton dynamic processes for a typical TADF emitter in OLEDs. Singlet and triplet excitons are generated in a 1 : 3 ratio depending on the spin degeneracy. F, P, ISC, IC, STA, TTA and RISC are abbreviations of fluorescence, phosphorescence, intersystem crossing, internal conversion, singlet–triplet annihilation, triplet–triplet annihilation, and reverse intersystem crossing.

Over the past two decades, the usage of phosphorescent materials has exploited the non-radiative triplet state by introducing heavy metal atoms, improving the overall electroluminescence (EL) internal quantum efficiency (IQE) of organic light emitting diodes (OLEDs) to 100%.^1,2^ However, more than the usage of noble metals like iridium (Ir) and platinum (Pt), which are expensive and non-renewable,^3^ the problem facing phosphorescent OLEDs (PH-OLEDs) is severe triplet–triplet annihilation (TTA) and triplet–polaron annihilation (TPA) at high brightness,^4^ which are fatal to practical PH-OLED application, not least because the EQE at luminances of 100 cd m^−2^ (display relevant) and 1000 cd m^−2^ (illumination relevant) is of great importance.^5^ An alternative approach for 100% IQE in OLEDs is utilizing thermally activated delayed fluorescence (TADF) materials.^6^ 75% of the electric-generated triplet excitons can be up-converted to the radiative singlet state to yield delayed fluorescence thanks to a very small Δ*E*_ST_.^7–9^ Analogous to PH-OLEDs, however, the EQEs of TADF-OLEDs at high brightness are also subjected to reduction by a large margin.^10,11^ Except for TTA, a higher singlet exciton density than Ir-based PH-OLEDs from an efficient RISC process produces a non-negligible STA.^12,13^ These superimposed factors make the reduced efficiency roll-off of TADF-OLEDs challenging on the march towards future high brightness applications (Scheme 1).

The short excited state lifetime of TADF materials (*τ*_TADF_) is important to reduce the efficiency roll-off of TADF-OLEDs by suppression of the TTA and STA.^4^ For TADF emitters, the rate constants of fluorescence (*k*_F_), intersystem crossing (*k*_ISC_), reverse intersystem crossing (*k*_RISC_), internal conversion (*k*_IC_), and non-radiative decay from the triplet state (*k*^T^_nr_) play roles in exciton dynamic processes and could govern *τ*_TADF_. Given that some of these factors are also involved in the EQE annihilation model of TADF-OLEDs (2c, ESI†),^13^ it is reasonable to deduce that tuning these factors *via* chemical modification could eventuate in various EQE roll-off curves even in exactly the same device architecture. Notably, it can be complicated to comprehend and design materials taking all factors into account, and the critical point for tuning *τ*_TADF_ remains unclarified. Therefore, selectively tuning these factors does help to find out the key to govern *τ*_TADF_, and ultimately not only assists in promoting efficiencies of TADF-OLEDs at practically relevant brightness, but can be used for reference towards material design in other specific fields where excited lifetime tuning is required.

To handle this, the governing of these factors should be linked to molecular design. To our knowledge, *k*_RISC_ is proportional to exp(−Δ*E*_ST_/*k*_B_*T*),^8^ where *k*_B_ denotes the Boltzmann constant and *T* denotes temperature. Δ*E*_ST_ is proportional to the orbital overlap (S_if_) of the initial (*Φ*_i_) and final (*Φ*_f_) state wavefunctions of the S_1_ transition. Meanwhile, *k*_F_ is related to the transition integral (H_if_) and is roughly proportional to S_if_.^14,15^ Therefore, *k*_F_ and *k*_RISC_ cannot be cut apart for TADF emitters, because a large *k*_F_ originating from a large frontier orbital overlap could enlarge Δ*E*_ST_ and reduce *k*_RISC_. A convenient approach to tune *k*_F_ and *k*_RISC_ is introducing twisted intramolecular charge transfer (TICT) systems with different dihedral angles between electron donating and withdrawing moieties.^7,9^ Enlarging the dihedral angles between D–A systems minimizes the transition integral, resulting in reduced *k*_F_ and Δ*E*_ST_ and *vice versa*. For emitters without heavy atoms, metals or halogens, *k*_ISC_ can be tuned using emitters with π–π* or n–π* transition character, for the latter ones facilitate the forbidden transition from the singlet to triplet excited state that violates spin conservation to enhance *k*_ISC_.^16–18^ For *k*_IC_, utilizing rigid chemical moieties and compact molecular structure design can remarkably suppress non-radiative channels. Notably, non-radiative processes in the triplet state are much slower than others given that *T*_1_ is relatively stable for pure aromatic compounds, and the influence of *k*^T^_nr_ can be temporarily ignored for simplicity.^19^ By the aforementioned criteria, molecules exhibiting specific characters can be designed.

**Fig. 1:**
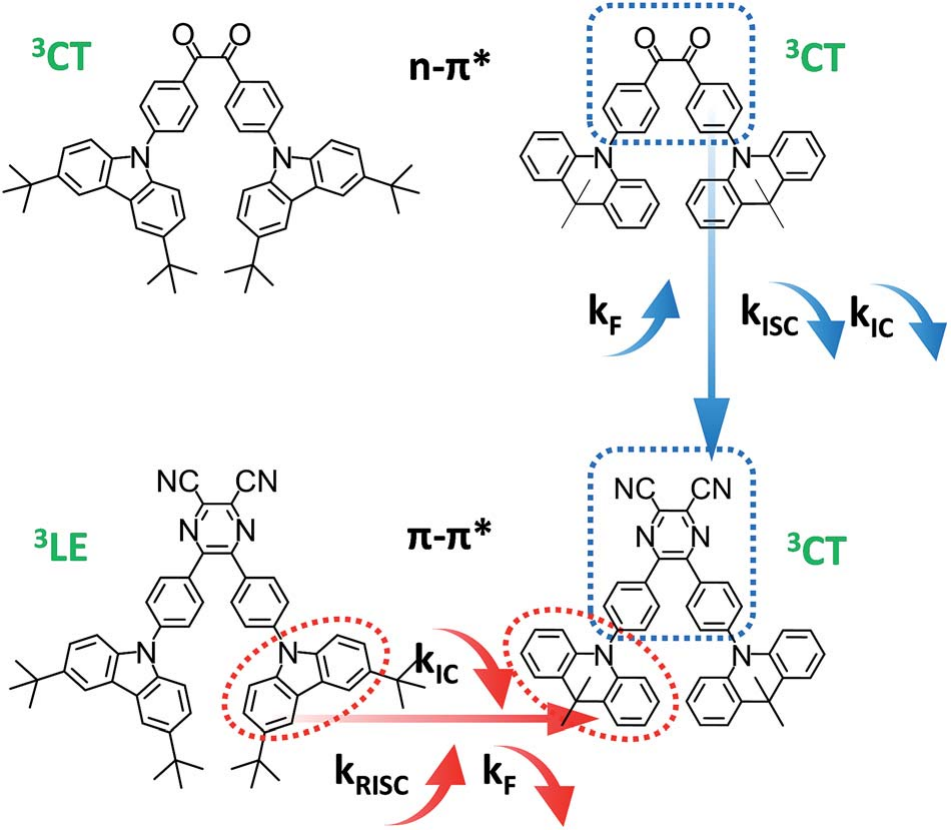
Molecular structures of the investigated compounds and molecular design for tuning *k*_F_, *k*_IC_, *k*_ISC_ and *k*_RISC_.

In this work, instead of starting from host engineering or device architecture management, we design four typical TADF emitters to elaborate the connection among *τ*_TADF_ governing, device efficiency roll-off control and molecular design. Among the diverse chemical moieties, carbonyl-type compounds are chosen for their special n–π* transition features, which inevitably renders a small *k*_F_ and a significant ISC process.^17,20–22^ Along this line of thinking, the benzil (DC) moiety is selected. On one hand, its orthogonal n and π* orbitals diminishes the exchange integral of the highest occupied molecular orbital (HOMO) and the lowest unoccupied molecular orbital (LUMO), enabling a fairly small Δ*E*_ST_ around 0.1–0.2 eV.^14,23^ Additionally, alternation of the electronic configuration can be realized *via* simple modification of the DC core, affording another representative radiative conducive π–π*-favoured constituent. The replacement of benzil by dicyano-pyrazine (PyCN) offers typical fluorophores with a large *k*_F_, reduced *k*_IC_ and *k*_ISC_.^24,25^ Aside from the acceptor cores, the donors are fixed on TC (3,6-di-*tert*-butylcarbazole) and ACR (9,10-dihydroacridine) moieties, respectively. The six-membered ACR ring induces enlarged D–A twisting compared with the five-membered TC heterocycle,^9^ leading to a reduced Δ*E*_ST_ and enhanced molecular rigidity for DC–ACR and PyCN–ACR. The aforementioned design thinking embodies four distinct and representative types of molecules as part of a large class of TADF emitters with analogous characters. The influence of molecular design on governing *τ*_TADF_ by tuning *k*_F_, *k*_ISC_, *k*_IC_ and *k*_RISC_ are investigated thoroughly by examining the photophysical properties and quantum chemical simulations. Lastly, OLEDs are fabricated to ascertain the affiliation between molecular design and device performance concerning efficiency roll-off (Fig. 1).

## Results and discussion

### Computation simulation

In order to demonstrate the influence of molecular design on governing *τ*_TADF_ by tuning *k*_F_, *k*_ISC_, *k*_IC_ and *k*_RISC_, comprehensive pictures of the molecules should be provided. Through adopting the appropriate methodology, density functional theory (DFT) and time-dependent density functional theory (TD-DFT) simulations can provide relatively reasonable descriptions of the investigated molecules.^26–28^ Initially, the ground state (S_0_) geometries of the four emitters were optimized at the B3LYP/6-31G* level.^28,29^ Different from the S_0_ geometry, S_1_ geometries for ICT molecules strongly depend on the exchange–exchange (XC) functional.^30^ Besides, selection of a proper functional to depict the vertical absorption transition energy (*E*_VA_) and emission energy (*E*_VE_) is required, because non-hybrid functionals neglect long-range coulombic attraction and underrate the CT transition energy while pure Hartree–Fock (HF) functionals suffer from electron correlation problems and overrate the excited energy.^31^ Lastly, the configuration interaction (CI) description of the S_1_ transition on coupling between the charge transfer excited state (CT) or locally excited state (LE) also relies on the XC functional.^9^ To simplify the simulation, a semi-empirical approach using experimental *E*_VA_(S_1_) to find the optimal percentage of the HF fraction (OHF) into functionals was adopted. Different HF% functionals were employed to compute *E*_VA_(S_1_) based on the optimized S_0_ geometry. HF% was plotted against *E*_VA_(S_1_) and the relationship was established (Fig. S7†). The optimized HF% (OHF) was determined to be close to 42% (BMK) by fitting the simulated *E*_VA_(S_1_) with well-defined CT absorption peaks from the ultraviolet-visible (UV-vis) spectroscopy measurement of all molecules.^32^ As S_1_ is sensitive towards XC functionals and solvent effects, it is necessary to optimize the S_1_ geometries, calculate *E*_VE_ and analyze the configuration interaction (CI) description at the BMK/6-31G* level utilizing the polarized continuum model (PCM) and so potential deviation can be minimized.^33,34^

**Scheme 2:**
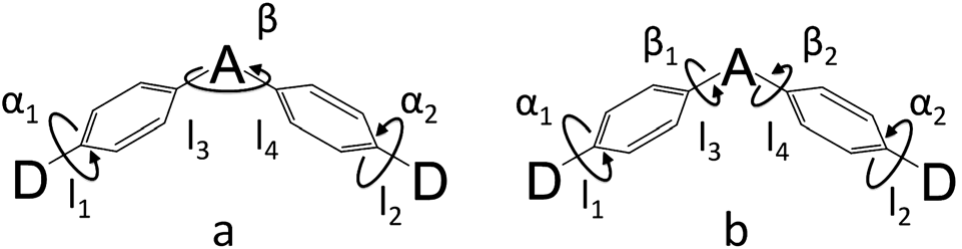
Two models for donor–π–acceptor–π–donor (D–π–A–π–D) molecules ((a) for DC–TC and DC–ACR, (b) for PyCN–TC and PyCN–ACR). *α* and *β* denote dihedral angles. *l*_1_ and *l*_2_ denote the bond lengths between the donor and phenyl bridge, while *l*_3_ and *l*_4_ denote the bond lengths between the acceptor and phenyl bridge.

**Fig. 2:**
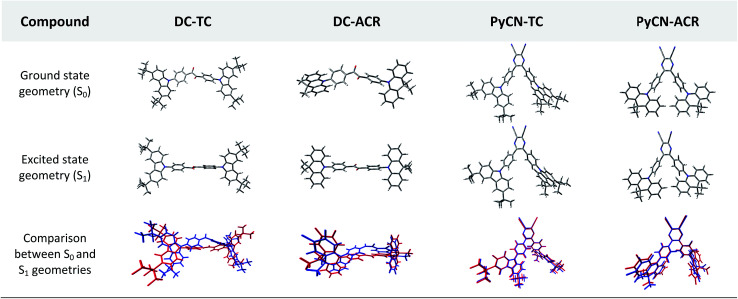
Front views of the DFT-optimized ground state geometries from B3LYP/6-31G* in the gas phase and excited state geometries from BMK/6-31G* in toluene using the PCM model for the four compounds. The bottom geometries are intuitive pictures comparing the S_0_ (blue) and S_1_ (red) geometries with minimum root-mean-square-deviation (RMSD).

**Fig. 3:**
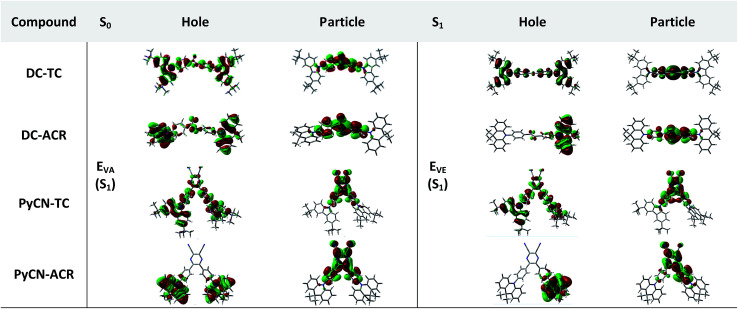
Natural transition orbital (NTO) analyses of the four investigated molecules. Hole and particle distributions of the HONTO and LUNTO of the S_0_ and S_1_ geometries are presented for intuitive comparison.

The simulated results of the four D–π–A–π–D compounds can be divided into two categories (Scheme 2). For the S_0_ and S_1_ geometries, detailed parameters including the bond length (*l*) and dihedral angle (*α* or *β*) are listed in Table 1. To visualize the frontier orbital transition of all compounds, natural transition orbital (NTO) analyses in view of singular value decomposition of the one-particle transition density matrix were carried out to comprehend the electron transitions, as NTO analyses provide fewer orbital pairs with maximum eigenvalues and simplify our interpretation.^35,36^ The geometries and NTOs of the four molecules are presented (Fig. 2 and 3), respectively.

**Table 1:** Calculated twisting angles (*α*_1_, *α*_2_ and *β*_1_, *β*_2_), bond lengths (*l*_1_, *l*_2_, and *l*_3_, *l*_4_), transition energies (*E*_VA_ and *E*_VE_ based on OHF functionals), and oscillator strengths (*f*_VA_ and *f*_VE_ based on OHF functionals) of all investigated molecules in the gas phase in S_0_-optimized geometries and in solvents in S_1_-optimized geometries

	S_0_ geometry						S_1_ geometry					
Compound	*α*_1_/*α*_2_ (°)	*β* or *β*_1_/*β*_2_ (°)	*l*_1_/*l*_2_ (Å)	*l*_3_/*l*_4_ (Å)	*E*_VA_(S_1_) (eV)	*f*_VA_	*α*_1_/*α*_2_ (°)	*β* or *β*_1_/*β*_2_ (°)	*l*_1_/*l*_2_ (Å)	*l*_3_/*l*_4_ (Å)	*E*_VE_(S_1_) (eV)	*f*_VE_
DC–TC	47.4/47.7	44.8	1.41/1.41	1.48/1.48	3.23	0.7712	50.0/50.3	3.0	1.41/1.41	1.50/1.50	2.80	0.5561
PyCN–TC	47.2/48.5	32.9/34.4	1.41/1.41	1.48/1.48	2.85	0.3632	50.0/47.1	21.9/28.8	1.41/1.41	1.46/1.47	2.55	0.4677
DC–ACR	89.1/89.3	49.5	1.43/1.43	1.49/1.49	2.77	0.0002	86.8/86.5	5.8	1.43/1.43	1.49/1.49	2.06	0.0011
PyCN–ACR	89.3/89.3	35.1/35.1	1.43/1.43	1.48/1.48	2.59	0.0001	89.9/89.4	40.7/15.8	1.43/1.44	1.49/1.43	2.11	0.0022

In the S_0_ geometry, the six-membered ACR offers a large steric hindrance effect from repulsion between the adjacent hydrogen atoms and *α* = 89° was estimated, much larger than that between TC and Ph (around 47°). The benzil moiety twists and avoids forming a flat surface in S_0_. *β* between PyCN and the Ph bridge lies around 34° (Fig. S6†) and twists to avoid repulsion with the *ortho*-substituted Ph ring. For vertical electron excitation, the S_0_ to S_1_ transition of DC–TC locates at the benzil moiety and has a much smaller oscillator strength (*f* ≈ 0) than S_2_; the latter is regarded as S_1_ in the following discussion. The lowest unoccupied natural transition orbitals (LUNTOs) of DC–TC and PyCN–TC predominantly localize on the DC and PyCN cores and slightly delocalize over the adjacent Ph ring. The highest occupied natural transition orbitals (HONTOs) distribute mainly on both TC–Ph arms and a small amount on the acceptor plane. Owing to the enlarged *α*, DC–ACR and PyCN–ACR have small orbital overlap, with the HONTO localizing mostly on ACR and the LUNTO localizing on the DC and PyCN–2Ph centers. The decreased coplanarity between the donor and acceptor prohibits nitrogen lone pair electrons from delocalizing over the whole molecule. In view of the more limited HONTO and LUNTO overlap for DC–ACR and PyCN–ACR, a much smaller *f* can be anticipated than DC–TC and PyCN–TC.

In S_1_, the bond lengths (*l*_1_, *l*_2_, *l*_3_, *l*_4_) show rather small variations in comparison with the S_0_ geometry. However, *α* and *β* change significantly, suggesting molecular geometry relaxation happens in S_1_. *β* decreases from 44.8° to 3.0° for DC–TC and from 49.5° to 5.8° for DC–ACR. A similar trend is also observed for PyCN–TC, as both *β*_1_ and *β*_2_ show a slight decrease to induce a more conjugated acceptor plane. However, for PyCN–ACR, *β*_2_ decreases from 35.1° to 15.8° while *β*_1_ increases from 35.1° to 40.7°, which can be explained by a more compact geometry compared with PyCN–TC. To twist a rigid ACR–Ph arm inevitably enhances the steric hindrance of the other, which is forced to avert close interaction between the adjacent ACR–Ph arms. The images comparing the S_0_ and S_1_ geometries (Fig. 2) clearly illustrate a degree of deformation and qualitatively depict more enhanced rigidity with smaller discrepancy. The coplanarization of acceptors and the internal twist of donors can be interpreted by the minimum overlap rule, which drives more complete charge separation of the donor and acceptor so as to lower the CT state energy.^37^ The flexible benzil acceptor tends to elongate conjugation, and this and the slightly increased *α* both contribute to separate further the HONTO and LUNTO and weakens electronic coupling between the donor and acceptor.^38–40^ Compared with DC–TC and DC–ACR, more suppressed non-radiative deactivation processes can be expected for PyCN–TC and PyCN–ACR showing lower geometry deformation in S_1_.

In the optimal S_1_ geometries, NTO transitions analogous to those in S_0_ are observed for DC–TC and PyCN–TC but broken-symmetry effects are noted for DC–ACR and PyCN–ACR. Followed by geometry relaxation, the degrees of HONTO and LUNTO overlap for DC–TC and PyCN–TC still far exceed those of DC–ACR and PyCN–ACR, indicating a larger *k*_F_ for the former ones. It is noteworthy that despite partially overlapping centres on the benzil moiety for the HONTO and LUNTO of DC–ACR, the n–π* transition nature of the benzil moiety hardly contributes to the overall *f* value.^21,22^ A *f* value roughly equal to zero is observed for such a typical n–π* transition in both the S_0_ and S_1_ geometries of the DC center, explaining the negligible *f*_VA_ and *f*_VE_ for DC–ACR (Fig. S5 and Table S1†).

To interpret the energy level coupling relationship, calculation of zero–zero energy levels (*E*_0–0_) is critical. A systematic comparison of *E*_VA_(S_1_), *E*_VE_(S_1_), *E*_0–0_(^1^CT), *E*_0–0_(^3^CT), *E*_0–0_(^3^LE) and Δ*E*_ST_ is listed (Table S2†). According to the Franck–Condon principle, the average values of *E*_VA_(S_1_) and *E*_VE_(S_1_) approximate the *E*_0–0_ energy level by eqn (1) and reproduce the experimental data with small deviation (Fig. S7†).^41^

1 *E*_0–0_(^1^CT) = (*E*_VA_(S_1_) + *E*_VE_(S_1_))/2

**Scheme 3:**
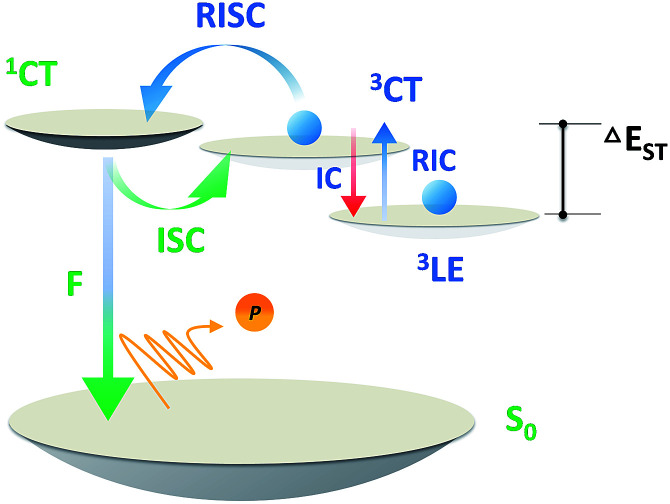
Exciton dynamic process for TADF emitters with and without a local triplet excited state (^3^LE). RIC denotes reverse internal conversion.

For analyzing the RISC process, coupling between the CT and LE triplet states should be carefully defined. The lower-lying ^3^LE state than the ^3^CT state ought to decrease the rate constants of the RISC process because of the slow reverse internal conversion (RIC) rate from the ^3^LE state to the ^3^CT state.^9^ Both *E*_0–0_(^3^CT) and *E*_0–0_(^3^LE) were computed independently based on eqn (2) and (3) (Scheme 3).^42^

2 *E*_0–0_(^3^CT) = *E*_0–0_(S_1_) − (*E*_VA_(S_1_, OHF) − *E*_VA_(S_1_, OHF)/*E*_VA_(S_1_, BLYP) × *E*_VA_(T_1_, BLYP))

3 *E*_0–0_(^3^LE) = *E*_VA_(T_1_)/(*E*_VA_(S_1_, OHF)/*E*_VA_(S_1_, BLYP)) − 0.09

Base on the calculated *E*_0–0_(^3^CT) and *E*_0–0_(^3^LE), the lower energy level turns out to be the lowest T_1_ state and Δ*E*_ST_ can be obtained. The reverse internal conversion (IC) rate is determined by the energy gap and the thermal equilibrium process between ^3^LE and the higher ^3^CT.^9^ A lower-lying ^3^LE state than the ^3^CT state impedes an efficient RISC process and enlarges Δ*E*_ST_.^43^ Triplet exciton generation at the ^3^LE state is hardly up-converted into the radiative ^1^CT state, which leads to a high triplet exciton density. Except for PyCN–TC, all the evaluated *E*_0–0_(^3^LE) are higher than *E*_0–0_(^3^CT). The *E*_0–0_(^3^LE) of PyCN–TC is lower than *E*_0–0_(^3^CT) by 0.31 eV and could be the predominant energy level forming triplet excitons under optical excitation or in EL devices. The difference between *E*_0–0_(^1^CT) and lowest T_1_ defines Δ*E*_ST_, which is proportional to the degree of orbital overlap (S_if_). With qualitative analyses of the overlap extent of HONTO and LUNTO, larger Δ*E*_ST_ values are calculated for DC–TC (0.21) and PyCN–TC (0.46) than DC–ACR (0.01) and PyCN–ACR (0.01) because the almost orthogonal dihedral angles between ACR and the acceptor cores induce a more separated HONTO and LUNTO. Due to the existence of the low-lying ^3^LE state, the calculated Δ*E*_ST_ of PyCN–TC reaches 0.46 eV, which is over twice as much as its chemical precursor DC–TC. Since similar *α* values were observed for DC–TC and PyCN–TC, the distinction for Δ*E*_ST_ can be explained by the different electronic configuration of the acceptor moiety. When the n–π* transition DC center is chemically modified into PyCN, the increased π–π* transition nature enhances the HONTO and LUNTO overlap, promoting a radiative transition process with a higher *k*_F_ and enlarged Δ*E*_ST_. As mentioned, additional attention should be stressed on the CT and LE coupling of the triplet state in molecular design because ^3^LE could intrinsically disable an efficient RISC process. The lowest ^3^CT and small ^1^CT/^3^CT energy splitting enables an unhindered RISC process, which leads to reduction of the triplet exciton population under optical or electrical excitation.

### Photo-physical properties

**Scheme 4:**
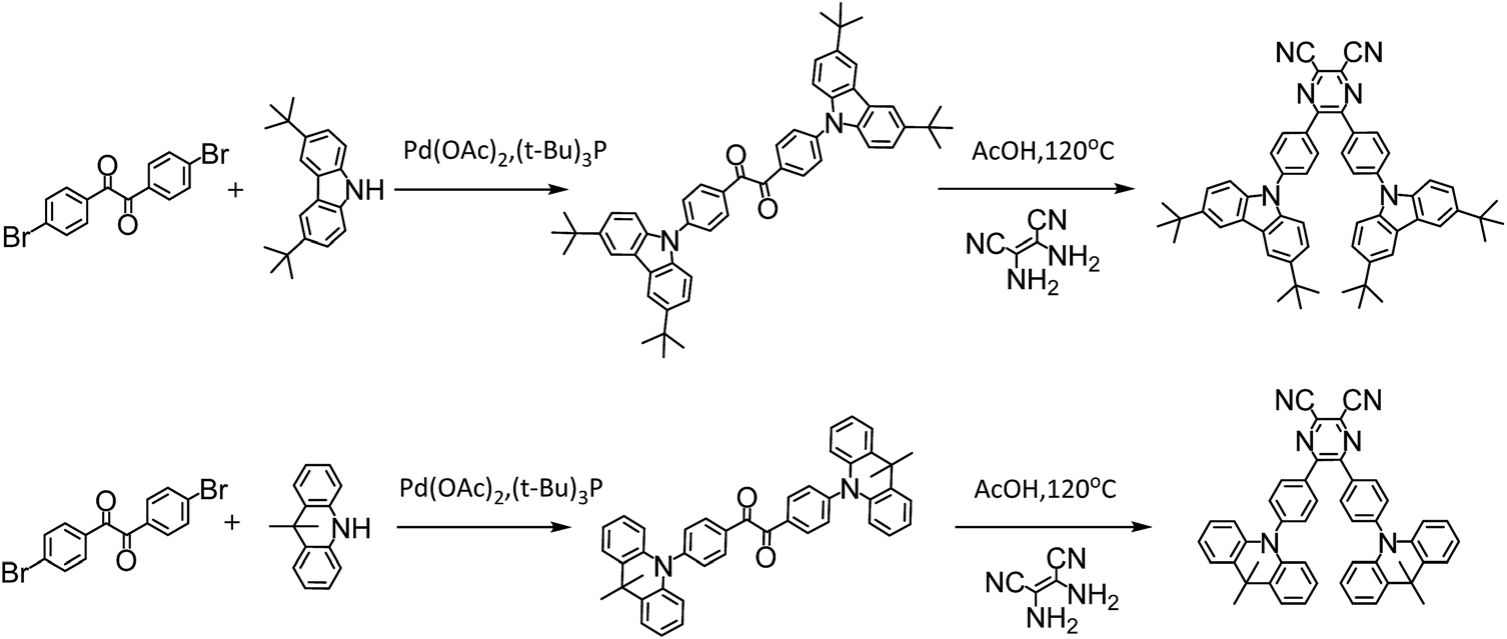
Synthesis routes for all compounds and reaction conditions.

**Fig. 4:**
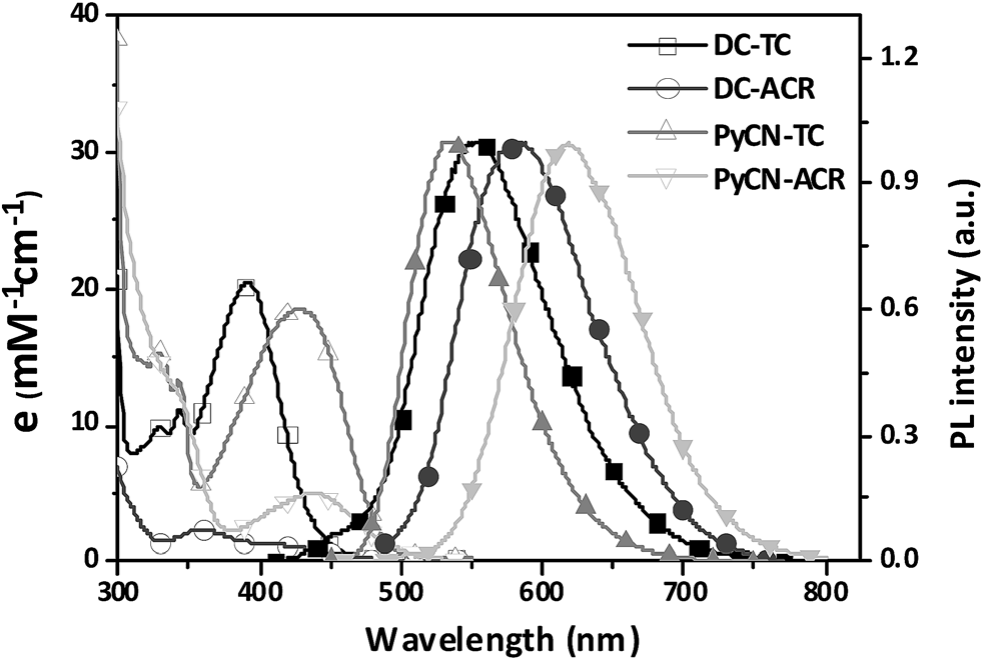
UV-vis absorption and PL spectra of the investigated molecules measured in toluene (DC–ACR in *n*-hexane) (0.05 mM) in air at room temperature.

The investigated compounds DC–TC and DC–ACR were synthesized from commercially available starting materials in a one-step convenient Buchwald–Hartwig C–N coupling reaction (Scheme 4). Based on DC–TC and DC–ACR, dicyano-pyrazine ring-closing products were easily produced followed by reactions with diaminomaleonitrile (DAMN) in acetic acid in high yields (>85%), making these compounds cost effective.^24,25^ Their photophysical properties were studied in diluted solutions and doped films. Broad ICT absorption and photoluminescence (PL) bands were observed. For DC–TC and PyCN–TC, the vertical absorption transition exhibits a larger molar extinction coefficient (*ε* = 18.0–21.0 mM^−1^ cm^−1^) than those of DC–ACR and PyCN–ACR (*ε* = 2.5–5.0 mM^−1^ cm^−1^). The large *ε* of DC–TC and PyCN–TC absorption can be understood by enhancement of the transition integral (H_if_) from large frontier orbital overlap. The solvatochromism effect on the absorption of all compounds was investigated (Fig. S10†). A fluctuating molar extinction coefficient intensity as well as drifting of the absorption peak position was noted. Except for a solvation effect on the Franck–Condon transition,^44^ diverse S_0_ molecular configuration distribution in various solvents can play a part. According to the DFT simulation geometry and the TD-DFT computed oscillator strength (*f*), the orthogonally connected ACR and Ph inevitably give rise to a forbidden HONTO to LUNTO transition (*f* ≈ 0). Nevertheless, a vertical transition absorption with moderate *ε* = 2.5–5.0 mM^−1^ cm^−1^ still can be detected, not to mention the moderate photoluminescence quantum yield (PLQY) of PyCN–ACR measured in the doped film state considering *k*_F_ is closely related to the *f* value of ICT vertical absorption (Fig. 4).^14^

**Fig. 5:**
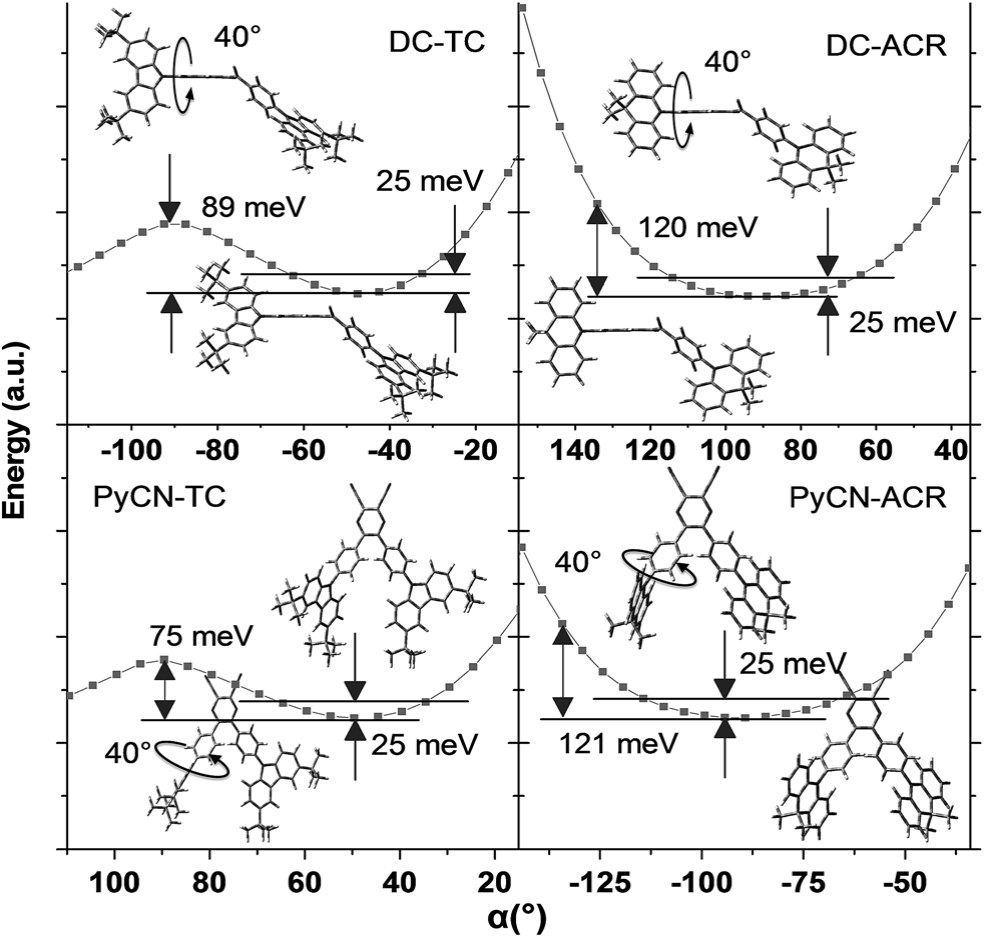
Flexible potential surface scanning on the energy of the ground state geometry at different twisted angles (*α*) in toluene from BMK/6-31G*. The two solid lines label the thermally activated energy (25 meV) at 300 K. The molecular insets represent the stationary point energy-optimized geometry (with *α* = 40° rotation) and the lowest energy-optimized geometry.

For greater understanding, flexible potential surface scans (full geometry optimization for each molecular conformation) were conducted for all molecules (Fig. 5). Due to symmetric double donor arms in the optimized S_0_ geometry, only one arm was selectively rotated for simplicity to explicate energy variation and molecular geometry distribution. Starting from the initial geometries, molecules were optimized (with Δ*α*_1_ = ±5° a step) to relax the irrational intramolecular interaction force, affording a more accurate vertical excitation energy and rotational energy barrier. Local energy maxima of DC–TC and PyCN–TC were obtained following a rotation Δ*α*_1_ = ±40°. The energy discrepancy between the minimized and local maximum energy turns out to be 89 meV for DC–TC and 75 meV for PyCN–TC. When undergoing the same twisting angle for DC–ACR and PyCN–ACR, the energy discrepancy enlarged to around 120 meV for both molecules. The more rigid ACR–Ph arm than TC–Ph accounts for this, and a steeper potential energy surface can be expected. Due to the large dihedral angle at the minimized energy geometry, the ACR–Ph arm is relatively stationary, which leads to the almost forbidden transition. However, ambient temperature (300 K) with 25 meV energy endows possible molecular rotation around Δ*α*_1_ = ±25°.^45,46^ The transition is more permissible because the more planar D–A conformation contributing to larger orbital overlap is found around the equilibrium conformation after activation by thermal energy. Theoretical absorption spectra of DC–ACR and PyCN–ACR were simulated based on OHF% functionals according to different twisting angles (Δ*α*_1_) using Multiwfn (Fig. S11†).^47^ When the twisted angle (Δ*α*_1_) increased from 5–25° (energy in the range of 25 meV), the *f* of ICT absorption asymptotically increases. In light of this, a moderate *k*_F_ and suppressed non-radiative process can be obtained simultaneously by the rigid but rotation-feasible ACR–Ph arms (Table 2).

**Table 2:** Thermal, electrochemical and photo-physical properties of the investigated molecules

Compound	*T*_d_ a/*T*_g_^b^ (°C)	IP/EA^c^/EA^d^ (eV)	*E*_g_ e/*E*_g_^f^ (eV)	*λ*_abs_ (nm) sol^g^	*λ*_PL_ (nm) sol^g^/film^h^	PLQY (%) sol^g^/film^h^
DC–TC	413/78	−5.56/N.A./−2.9	N.A./2.58	389	553/518	0.3 (0.6)/14.7 (19.0)
DC–ACR	387/98	−5.35/N.A./−2.84	N.A./2.51	411	584/532	0.8 (2.0)/11.5 (13.0)
PyCN–TC	403/N.A.	−5.56/−3.32/−3.26	2.24/2.30	429	532/534	53.8 (62.9)/28.6 (38.0)
PyCN–ACR	450/N.A.	−5.33/−3.18/−3.17	2.15/2.16	438	618/560	8.6 (16.4)/39.8 (43.0)

a Decomposition temperature (5% weight loss).

b Glass transition temperature.

c Ionization potential (IP) and electron affinity (EA) measured by cyclic voltammetry in 0.1 M *n*-Bu_4_NPF_6_ in CH_2_Cl_2_ : CH_3_CN (4 : 1) solution.

d EA was calculated by IP minus optical gap approximated by the absorption edge in toluene.

e Energy gap estimated from cyclic voltammetry measurement.

f Energy gap estimated from the absorption edge in toluene (DC–ACR in *n*-hexane).

g The longest peak wavelength of absorption and PL measured in toluene (*n*-hexane for DC–ACR) and absolute PL quantum yield (PLQY) evaluated using an integrating sphere in toluene (*n*-hexane for DC–ACR) before and after N_2_ bubbling (in parenthesis).

h PLQY of 7 wt% investigated molecules doped into CBP in air and N_2_ atmosphere (in parenthesis), respectively.

**Fig. 6:**
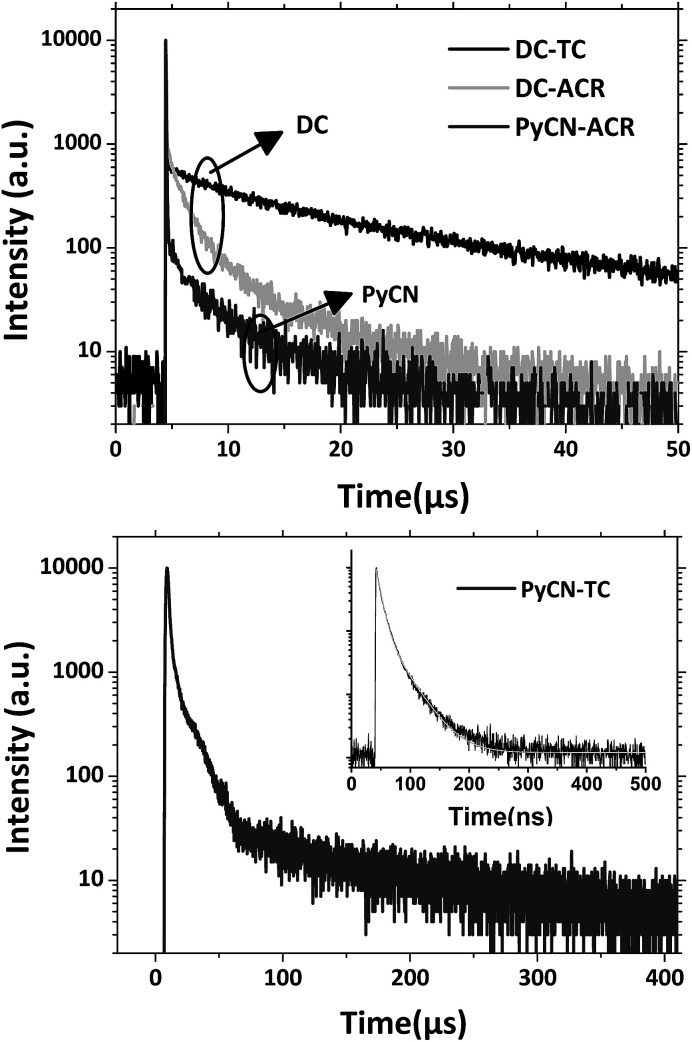
Transient PL decay spectra of the compounds doped into CBP films (7 wt%) measured with a fluorescence lifetime spectrometer (C11367-03, Hamamatsu Photonics) at room temperature in a N_2_ atmosphere.

For the photoluminescence (PL) spectra (Fig. 6 and S12†), when increasing the polarity of the solvent, a positive solvatochromism effect was observed.^48^ DC–TC and DC–ACR were hardly emissive while ring-locked PyCN–TC and PyCN–ACR exhibited higher PLQY in solution. After N_2_ treatment, the PLQYs of the four compounds in solution increase compared with those in air equilibrium solutions, implying that the dissolved oxygen quenches the triplet state excitons.^49^ As revealed in TD-DFT (Fig. 3), the less rigid DC–TC and DC–ACR undergo obvious conformation deformation, leading to a quick internal conversion (IC) process in S_1_. When DC was chemically modified into PyCN, the increased rigidity significantly suppresses the non-radiative decay process and a higher PLQY can be obtained in liquid media. To verify such effects on restricted molecules, the PL of the four compounds in different water : THF mixtures ratio was measured (Fig. S13†). Higher water fractions increase the extent of aggregation, enabling suppression of non-radiative channels.^50^ Evidently, the emission of all the compounds was enhanced with increased aggregation when a high fraction of water was added.

In addition, the character of the triplet emission was analyzed *via* phosphorescence spectra at 77 K. In good agreement with the TD-DFT analyses, the ^3^LE triplet state of PyCN–TC was experimentally confirmed. The phosphorescence spectra of PyCN–TC suggested a specific vibrational structure. For the other three, the TD-DFT-simulated lowest ^3^CT triplet states are verified by single ICT phosphorescence peaks. (Fig. S9†) Δ*E*_ST_ of all the compounds can be calculated and the order is DC–ACR (0.01 eV) > PyCN–ACR (0.08 eV) > DC–TC (0.13 eV) > PyCN–TC (0.43 eV), in good agreement with the quantum simulation results.

**Fig. 7:**
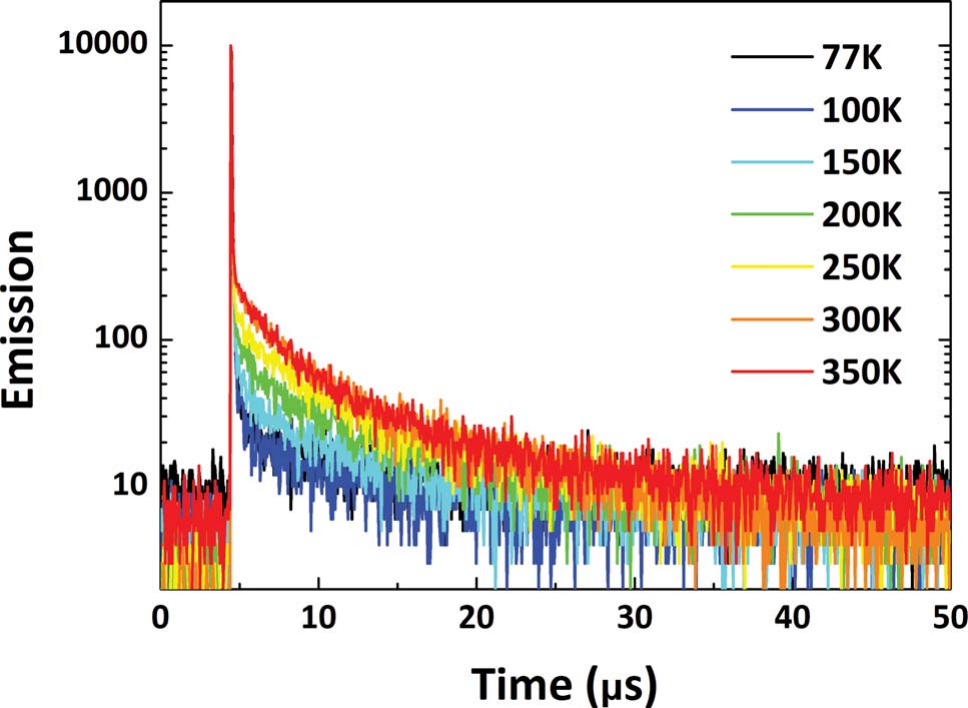
Transient PL decay curves for 1 wt% PyCN–ACR doped into CBP measured from 77 to 350 K in a N_2_ atmosphere.

**Scheme 5:**
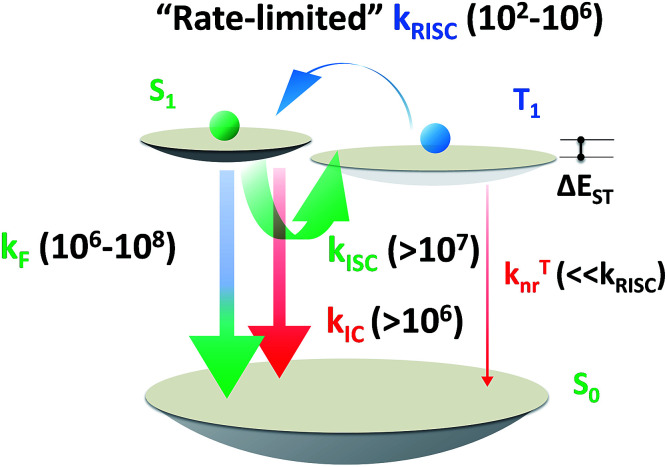
Order of magnitude of rate constants involved in exciton dynamic processes for TADF emitters. As shown, *k*_RISC_ becomes the rate-limited step during these processes for tuning the lifetime of the excited state (*τ*_TADF_). *k*_F_, *k*_ISC_, *k*_IC_ and *k*_RISC_ are on the orders of 10^6^ to 10^8^, >10^7^, >10^6^ and 10^2^ to 10^6^ respectively, as summarized by previous reports.^7–9,13,15,51–54,56,58,61,64^

To factually assess performance of the emitting materials in devices, the solid-state photo-physical properties of all the materials were investigated by doping them into 4,4′-bis(carbazol-9-yl)biphenyl (CBP). The doping concentration was controlled at a low level to avoid aggregation-induced quenching effects. The emission in the doped films blue-shifted by around 10–60 nm in contrast to that in solution, indicating that molecular reorganization and relaxation are hindered by the surrounding solid matrix with a reduced Stokes bathochromic shift (Fig. S14†).^15^ To verify the TADF nature of all the materials, temperature-dependent transient PL spectra were measured from 77 to 350 K (Fig. 7 and S15–S17†). The delayed fluorescence ratio of all the materials showed enhancement when the temperature was increased, then some of the fluorescence ratios were followed by a slight decrease as the temperature was further increased to 350 K. For PyCN–ACR, for example, the monotonically enhanced ratio of the delayed fluorescence from 9.7% to 60.5% firmly depicts the enhanced contribution of RISC from 77 to 300 K. Conversely, further increasing the temperature from 300 to 350 K reduced the delayed fluorescence ratio from 60.5 to 56.2%. Other than the suppressed IC process at low temperature, it competes significantly with RISC at high temperature and results in a reduced delay fluorescence ratio, which is one of the typical TADF characters.^51^ An analogous phenomenon was also observed for DC–TC, DC–ACR and PyCN–TC, putting all of them into the category of TADF materials (Scheme 5).

**Fig. 8:**
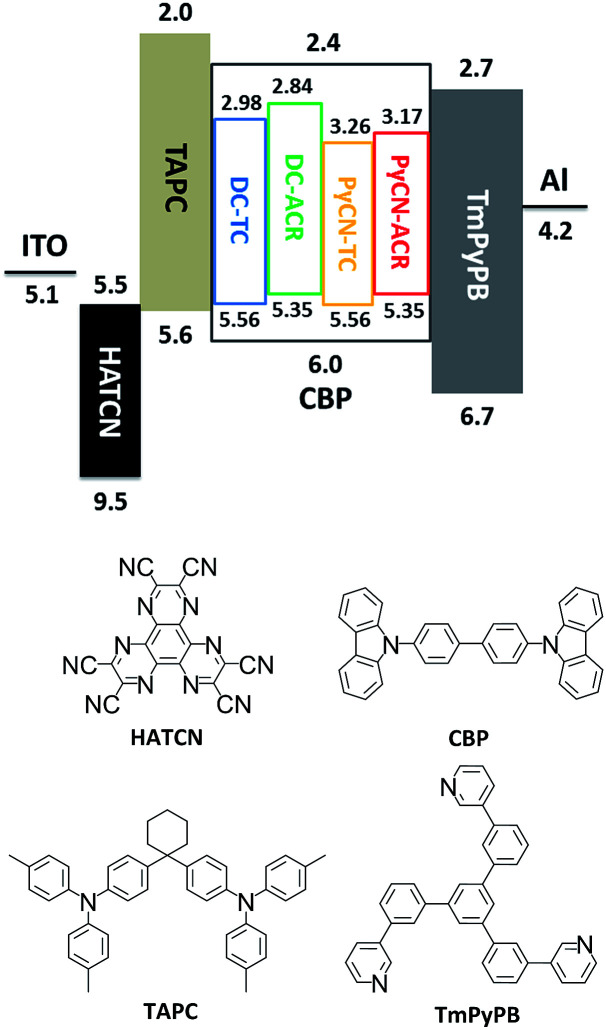
Energy level diagram and molecular structures of the materials for fabricating OLED devices. The HOMO and LUMO levels of the investigated molecules were estimated by cyclic voltammetry.

Based on the transient PL decay curve at 300 K in a N_2_ atmosphere (Fig. 8), the prompt (*Φ*_F_) and delayed (*Φ*_TADF_) components of the PLQY as well as the prompt decay lifetime (*τ*_p_) and delayed decay lifetime (*τ*_TADF_) were separated for successive calculation. *k*_F_, *k*_ISC_, *k*_RISC_ and *k*_IC_ were estimated using the following formulas.^8,15,52,53^

4 *k*_F_ = *Φ*_F_/*τ*_p_

5 *Φ*_ISC_ = 1 − *Φ*_F_

6 *Φ*_F_ = *k*_F_/(*k*_F_ + *k*_ISC_)

7 *k*_RISC_ = (*k*_p_*k*_d_*Φ*_TADF_)/(*k*_ISC_*Φ*_F_)

8 *Φ* = *k*_F_/(*k*_F_ + *k*_IC_)

As expected, the triplet yields (*Φ*_ISC_) of DC–TC (93%) and DC–ACR (97%) are superior to those of PyCN–TC (67%) and PyCN–ACR (80%). Optically generated S_1_ excitons are inclined to ISC into triplet states for DC–TC and DC–ACR because the n–π* transition of carbonyl derivatives favours an ISC process.^17^ In contrast, when DC was chemically modified into PyCN, *Φ*_ISC_ and *k*_ISC_ were reduced and an enhanced *k*_F_ was observed (PyCN–TC contrast to DC–TC, and PyCN–ACR contrast to DC–ACR, respectively). Owing to the similar *α* for DC–TC and PyCN–TC or DC–ACR and PyCN–ACR, the increased π–π* character induces a larger *k*_F_ for the latter pair. Additionally, when comparing the rigidity of DC–TC to PyCN–TC or DC–ACR to PyCN–ACR, lower *k*_IC_ values were observed for the latter owing to the more rigid PyCN core. Though PyCN–ACR shows a redder emission, the compact molecular structure results in the lowest *k*_IC_ among all the emitters. The *k*_RISC_ of DC–ACR and PyCN–ACR are an order of magnitude higher than that of DC–TC and PyCN–TC, indicating a more efficient RISC from T_1_ to S_1_ for the former pair. Interestingly, the delayed lifetimes of carbonyl-type DC–TC and DC–ACR differ greatly with analogous *Φ*_ISC_ ratios (over 90%). In this case, the shortening *τ*_TADF_ of DC–ACR should be ascribed to a more efficient RISC process, and an even larger *k*_F_ for DC–TC (0.56 × 10^7^) than DC–ACR (0.13 × 10^7^) was observed. For n–π*-favored DC–ACR and π–π*-favored PyCN–ACR with different *k*_ISC_ and *k*_F_ values, a shorter *τ*_TADF_ was still observed for the former, with smaller Δ*E*_ST_, reduced *k*_ISC_ and enhanced *k*_F_ contributing to the shortening of *τ*_TADF_.

Based on a parallel comparison, as *k*_F_, *k*_ISC_, *k*_IC_ and *k*_RISC_ all contribute to *τ*_TADF_ tuning, a large *k*_RISC_ plays the leading part. This can be interpreted by the “rate-limited effect” of the RISC process. For TADF emitters, the slow dissipation of triplet excitons and an inefficient RISC process can result in a rather long excited state lifetime. As *k*_F_, *k*_ISC_ and *k*_IC_ are larger than 10^6^ while *k*_RISC_ is from 10^2^ to 10^6^,^7–9,54,55^ the much smaller *k*_RISC_ becomes the rate-limited step in exciton dynamic processes. As the triplet state can be stable and its non-radiative process is much slower than RISC, the enhanced *k*_RISC_ becomes critical because the up-converted triplet excitons can be deactivated quicker radiatively or non-radiatively in the S_1_ state. According to the analyses, a much shorter *τ*_TADF_ can be expected for DC–ACR even with a large *k*_ISC_ and a small *k*_F_. For PyCN–TC, although the highest *k*_F_ and small *k*_ISC_ values are calculated, *τ*_TADF_ of PyCN–TC is much longer than the other three emitters considering it has the smallest *k*_RISC_. Based on the molecular design, we are able to figure that increasing *k*_RISC_ is the key to shortening *τ*_TADF_ (Table 3).

**Table 3:** Photo-physical data of the investigated molecules doped into CBP films (7 wt%) at room temperature

Compound	*λ*_em_ (nm)	*Φ*	*Φ*_F_	*Φ*_TADF_	*Φ*_ISC_	*τ*_p_ (ns)	*τ*_TADF_ a (μs)	*k*_F_ (×10^7^ s^−1^)	*k*_IC_ (×10^7^ s^−1^)	*k*_ISC_ (×10^7^ s^−1^)	*k*_RISC_ (×10^5^ s^−1^)	Δ*E*_ST_ (eV)
DC–TC	518	0.19	0.07	0.12	0.93	12.6	24.9	0.56	2.4	7.4	0.74	0.13
DC–ACR	532	0.13	0.03	0.10	0.97	22.8	3.0	0.13	0.9	4.3	11.4	0.01
PyCN–TC	534	0.38	0.33	0.05	0.67	25.2	210.5	1.31	2.1	2.60	0.011	0.43
PyCN–ACR	575	0.43	0.20	0.23	0.80	34.3	4.6	0.58	0.7	2.32	3.13	0.08

a Average lifetime calculated by *τ*_av_ = Σ*A*_i_*τ*_i_^2^/Σ*A*_i_*τ*_i_, where *A*_i_ is the pre-exponential for lifetime *τ*_i_.

### OLED characterization

After detailed investigation on the emitting properties and energy level relationships of the four TADF molecules, differences in the electroluminescence performance is investigated for the four materials. To reduce variants, the four TADF materials were employed in the same device architecture. Given that the carrier transport abilities of the investigated guest emitters have effects on the carrier balance and could influence the roll-off character of the devices, the doping concentration of the four TADF emitters were controlled at a low level (1 wt% for DC–ACR and PyCN–ACR, 5 wt% for DC–TC and PyCN–TC) but assuring complete energy transfer from host to guest. Consequently, the device efficiency roll-off can approximately be linked to the molecular characteristics. The fabricated device architecture is indium tin oxide (ITO)/HATCN (5 nm)/TAPC (20 nm)/*x* wt% emitter in CBP (35 nm)/TmPyPB (55 nm)/LiF (1 nm)/Al (100 nm), in which the dipyrazino[2,3-*f*:20,30-*h*]quinoxaline-2,3,6,7,10,11-hexacarbonitrile (HATCN), (1,1-bis(4-(*N*,*N*-di(*p*-tolyl)-amino)-phenyl)cyclohexane) (TAPC), 1,3,5-tri(*m*-pyrid-3-yl-phenyl)benzene (TmPyPB) and LiF layers play the roles of hole injection, hole transport and electron blocking, electron transport and hole blocking and electron injection layers, respectively.^56^ An energy level diagram is displayed in accordance with the cyclic voltammetry (CV) results (Fig. 8 and S23†).^4^ Unlike the inefficient energy transfer from host to guest under optical excitation (Fig. S14†), suppressed CBP host emission around 400 nm was noted in the EL spectra and additional energy capture channels should be considered. As the HOMO and LUMO of all guest molecules are shallower and deeper than that of CBP, relatively equilibrium hole and electron capture abilities can be obtained.^57,58^ Carriers can be trapped directly into the guest instead of solely transferring the energy *via* long-range Förster or short-range Dexter transfer, leading to complete energy transfer from host to guest in contrast to those under optical excitation (Fig. S14†).^59,60^

**Fig. 9:**
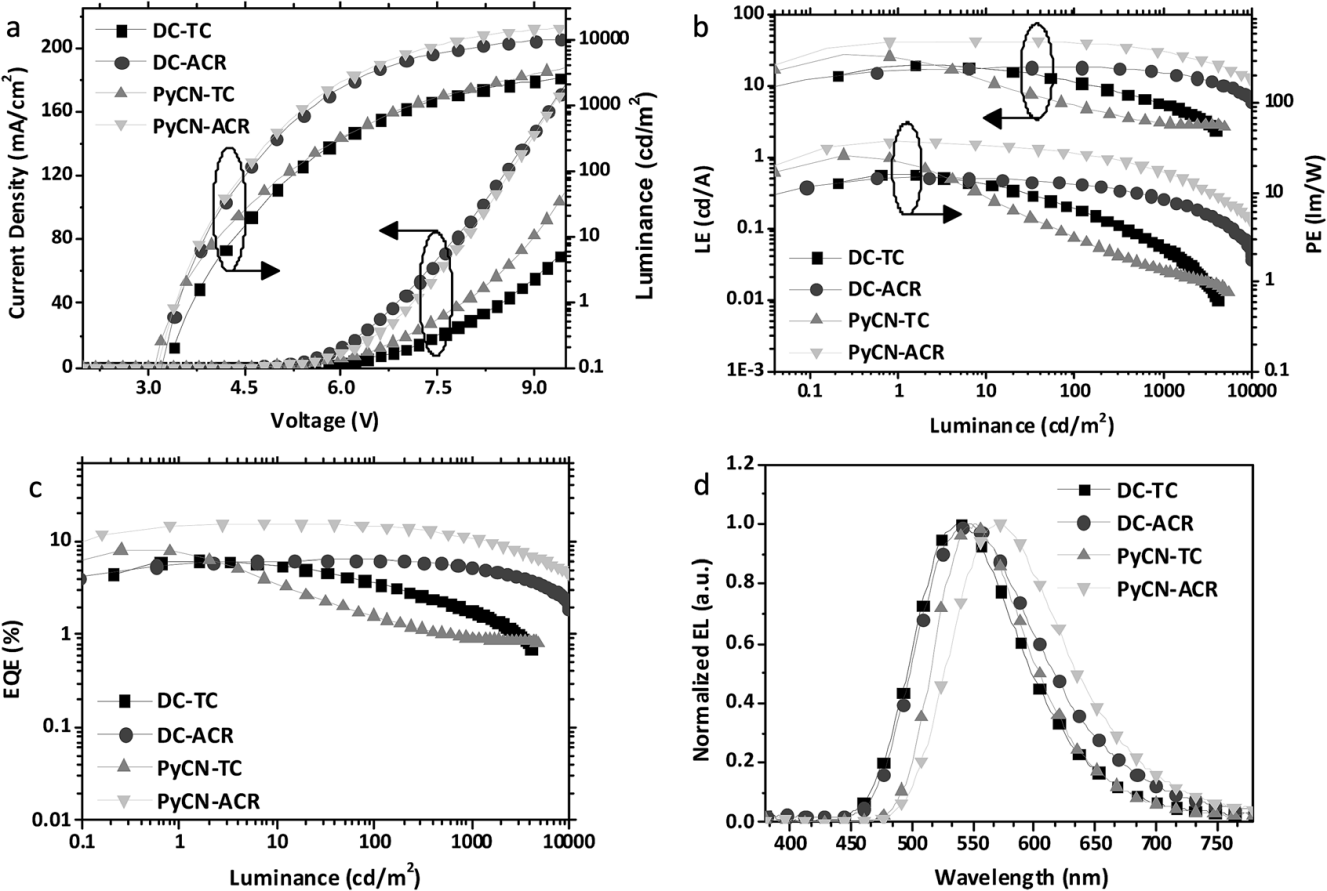
(a) Current density–voltage–luminance (*J*–*V*–*L*), (b) luminance efficiency–luminance–power efficiency (LE–*L*–PE), (c) EQE–luminance characteristics, and (d) EL spectra at 10 mA cm^−2^ of the devices based on the investigated molecules.

Current density–voltage–luminance (*J*–*V*–*L*), luminance efficiency–luminance–power efficiency (LE–*L*–PE), EQE–luminance (EQE–*L*) characteristics and EL spectra are listed in Fig. 9. From the EL spectra, emission peaks from greenish-yellow to orange locating at 538 nm for DC–TC, 546 nm for DC–ACR and PyCN–TC, and 572 nm for PyCN–ACR are observed. The turn-on voltages of the four materials are about the same at 3.4 to 3.5 V, detected at the luminance of 1 cd m^−2^. The maximum EQEs of the four devices are 6.2% (DC–TC), 6.3% (DC–ACR), 8.1% (PyCN–TC) and 15.6% (PyCN–ACR). Applications of benzil derivatives in OLEDs have been deemed to be impractical due to their poor PLQY. However, the achieved EQEs exceeding the 5% theoretical limitation of traditional fluorophores suggests the efficient utilization of 75% triplet excitons generated under electric excitation.

Different degrees of efficiency roll-off at high current density were observed, eliminating the possibility of a TTA mechanism leading to the high EQE. Among the four emitters, PyCN–TC exhibits the largest Δ*E*_ST_ and ^3^LE triplet state, eventuating in *τ*_TADF_ exceeding 200 μs. The hardly up-converted T_1_ exciton contributes less to delayed fluorescence and leads to a high triplet density population even with a high *k*_F_ and a small *k*_ISC_. The most significant efficiency roll-off was observed with only 23% and 11% of EQE_max_ remaining at luminances of 100 and 1000 cd m^−2^ respectively.

**Fig. 10:**
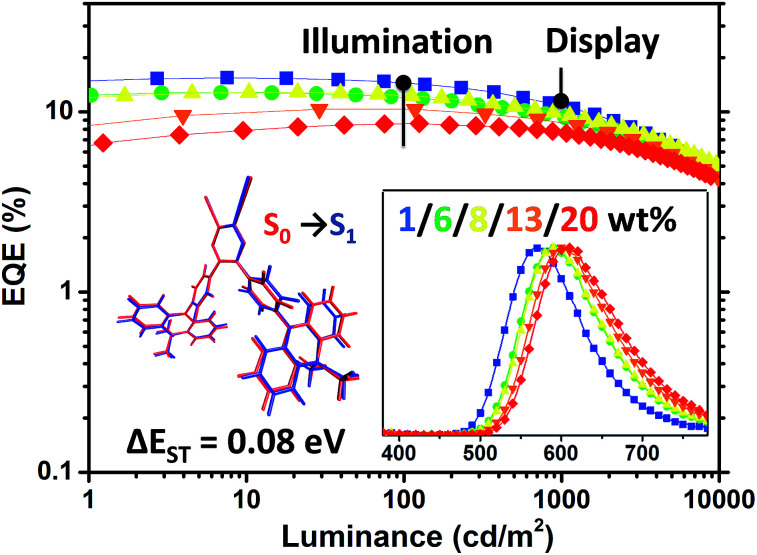
EQE–luminance curves of the OLED devices based on PyCN–ACR at various doping concentrations in a structure of ITO/HATCN (5 nm)/TAPC (20 nm)/CBP: *x* wt% PyCN–ACR (35 nm)/TmPyPB (55 nm)/LiF (1 nm)/Al. Inset: (left) small geometry deformation in the excited state and Δ*E*_ST_ for PyCN–ACR are responsible for highly efficient and low roll-off orange-red TADF-OLEDs; (right) EL spectra of the corresponding devices at 10 mA cm^−2^ with different doping concentrations of PyCN–ACR in CBP.

Towards DC–TC, DC–ACR and PyCN–ACR with the lowest ^3^CT triplet states, efficiency roll-off can also be linked to *τ*_TADF_. As mentioned, the enhanced *k*_RISC_ effectively shortens *τ*_TADF_, endowing reduced triplet exciton density for suppressed STA and TTA. For DC–TC, owing to the larger Δ*E*_ST_ than DC–ACR and PyCN–ACR, a much longer *τ*_TADF_ increased the ISC–RISC cycles between radiative S_1_ and non-radiative T_1_, resulting in a remarkable efficiency roll-off with only 37.1% of the EQE_max_ remaining at a luminance of 1000 cd m^−2^. In contrast, a well-controlled roll-off was achieved for DC–ACR with the shortest *τ*_TADF_ among four emitters, exhibiting 100% and 81% of the EQE_max_ at luminances of 100 and 1000 cd m^−2^ respectively. In spite of the close *Φ*_ISC_ ratio for DC–TC and DC–ACR and the even larger *k*_F_ for the former, an enhanced RISC process plays the dominant role in reducing the efficiency roll-off for TADF-OLEDs. For PyCN–ACR with a larger *k*_RISC_ than DC–TC, a lower device efficiency roll-off is also observed at practical brightness. Note that different doping concentrations of the guest could influence the efficiency roll-off of the device; devices with the same guest doping concentration (5 wt%) were also fabricated for comparison (Fig. S20†). For TADF-OLEDs using DC–ACR and PyCN–ACR as the emitters, the tendency is consistent with our analyses. Emitters with a large *k*_RISC_ tended to exhibit low efficiency roll-off character at practical brightness (Fig. 10).

Further information on the mechanism of large *k*_RISC_ for reduced efficiency roll-off can be found by fitting the EQE–*J* curve with the EQE roll-off model of TADF-OLEDs (Fig. S22†). A high singlet exciton concentration from efficient RISC, STA and TTA processes can be the dominant source for exciton annihilation.^13,61,62^ For a steady-state current, the model EQEs are fitted and afford the rate constants of STA (*k*_ST_) and TTA (*k*_TT_). Very small HONTO and LUNTO overlap for PyCN–ACR and DC–ACR renders a small *f* and the rate constant of Förster energy transfer-type STA could be low. Though the calculated *k*_TT_ are two magnitudes smaller than *k*_ST_, the triplet density can be over three orders higher than the singlet density and TTA could aggravate the efficiency roll-off of the device.^13,63^ In good agreement of our analyses, the *k*_ST_ and *k*_TT_ of DC–ACR and PyCN–ACR are much smaller than those of the other two emitters with longer *τ*_TADF_, indicating more suppressed STA and TTA for OLEDs using emitters with a large *k*_RISC_ and a short *τ*_TADF_.

Notably, the 1 wt% PyCN–ACR in CBP device exhibits orange EL at 572 nm. The slightly bathochromic shift (12 nm) with respect to the PL in the doped film implies geometry relaxation in the solid matrix. When the doping concentration was adjusted from 1 wt% to 20 wt%, a red-shift as large as 34 nm was observed. Under an electric field, TADF molecules with a large dipole moment tend to polarize in the host medium. Such a “solvatochromic” shift in the solid matrix can be observed when TADF emitters are doped into bipolar hosts or the doping concentration of the guest is increased.^64,65^ A low efficiency roll-off was noted for all the devices with different doping concentrations. The maximum efficiency roll-off turned out to be 38.8% at 1000 cd m^−2^ for 1 wt% PyCN–ACR doped into CBP. Especially for the devices with doping concentrations of 8 wt% and 13 wt%, EQEs of 12.4% and 10.3% at a luminance of 100 cd m^−2^ as well as EQEs of 9.9% and 8.8% at a luminance of 1000 cd m^−2^ were achieved. The roll-off was less than 25% and 15% and emission peaks were located at 590 and 600 nm, respectively. Even at a luminance of 10 000 cd m^−2^, EQEs as high as 5.1% and 4.4% were maintained. For PyCN–ACR, the rotation-feasible ACR–Ph arms, enhanced π–π* feature and compact molecular geometry endow a moderate *Φ* and restricted non-radiative channel for orange-red emitters with a narrow band gap. In combination with the high *k*_RISC_, a low efficiency roll-off and highly efficient orange-red device employing PyCN–ACR can be realized, achieving the best EQE of orange-red TADF-OLEDs at application relevant brightness (100/1000/10 000 cd m^−2^) without employing host management.

## Conclusions

*Via* molecular design, the effects of *k*_F_, *k*_ISC_, *k*_IC_ and *k*_RISC_ on *τ*_TADF_ governing were investigated based on four representative TADF emitters. *τ*_TADF_ can be effectively shortened by enhanced RISC (small Δ*E*_ST_) even with a small *k*_F_ and a large *k*_ISC_. As RISC is slower than other exciton dynamic processes and T_1_ is relatively stable, it becomes the rate-limited step in shortening *τ*_TADF_ (“rate-limited effect” of the RISC process). When TADF materials with short *τ*_TADF_ are employed in OLEDs, more suppressed efficiency roll-off at practical relevant brightnesses (100/1000/10 000 cd m^−2^) is observed. The shortened *τ*_TADF_ indicates a more efficient RISC process and eventuates in reduced triplet exciton density for suppressed STA and TTA.

Following the aforementioned guidelines, TADF materials should be carefully designed to govern *τ*_TADF_. In light of the critical role of *k*_RISC_, factors concerning tuning Δ*E*_ST_ can effectively alter *τ*_TADF_. One of the approaches is tuning the D–A strength of the emitters for enhanced ICT, which can render small frontier orbital overlap and reduce Δ*E*_ST_. A more promising way is tuning the dihedral angles between electron donating and withdrawing moieties in TICT molecular systems. The enlarged dihedral angle induces separated HOMO/LUMO overlap while a flat D–A structure does the opposite. The former case reduces Δ*E*_ST_ and effectively shortens *τ*_TADF_, giving these materials potential for fabricating low efficiency roll-off OLED devices. The latter case can elongate *τ*_TADF_, making these materials promising for ultra-long lifetime delayed fluorescence applications such as bio-imaging,^66^ oxygen sensors^67^ or anti-fake,^68^*etc.* Finally, tuning ^3^LE and ^3^CT coupling also helps to tune *τ*_TADF_. For TADF-OLEDs, materials with ^3^LE can be detrimental for shortening *τ*_TADF_ because the additional RIC process makes RISC tougher. OLEDs using such emitters exhibit significant roll-off, and a low EQE is expected at high brightness. Chemical moieties for fabricating TADF emitters should not have low triplet states to make sure that the locally excited state lies above the CT channel.

## Supplementary Material

SC-007-C6SC00542J-s001
