# Supplementary material for: “Rate-limited effect” of reverse intersystem crossing process: the key for tuning thermally activated delayed fluorescence lifetime and efficiency roll-off of organic light emitting diodes
Source: Chem Sci. 2016 Mar 15;7(7):4264–75. doi: 10.1039/c6sc00542j (PMC6013828; doi:10.1039/c6sc00542j)
Supplement: SC-007-C6SC00542J-s001 [file SC-007-C6SC00542J-s001.pdf]

Electronic Supplementary Material (ESI) for Chemical Science.
This journal is © The Royal Society of Chemistry 2016

Supporting Information

"Rate-Limited Effect" of Reverse Intersystem Crossing Process: the Key for Tuning Thermally Activated Delayed Fluorescence Lifetime and Efficiency Roll-Off of Organic Light Emitting Diodes

Xinyi Cai, Xianglong Li, Gaozhan Xie, Zuozheng He, Kuo Gao, Kunkun Liu, Dongcheng Chen, Yong Cao, Shi-Jian Su*

State Key Laboratory of Luminescent Materials and Devices and Institute of Polymer Optoelectronic Materials and Devices, South China University of Technology, Guangzhou 510640, China

*Corresponding author. E-mail: mssjsu@scut.edu.cn

Contents

1. Experimental Section
2. Supplemental Tables and Figures
    a) Synthesis and Characterization
    b) Quantum Chemical Computation
    c) Photoluminescence Measurements
    d) Device Fabrication and Measurements
    e) TTA and STA roll-off model simulation
    f) Thermal Properties
    g) Cyclic Voltammetry
3. References
4. Computation Geometry Data

1. Experimental Section

General. ^{1}H and ^{13}C NMR spectra were recorded on a Bruker NMR spectrometer operating at 600 and 150 MHz, respectively. All data were recorded in deuterated chloroform (CDCl3) solution at room temperature. Mass spectra were obtained using a JEOL JMS-K9 mass spectrometer. Thermogravimetric analyses (TGA) were performed on Netzsch TG 209 under nitrogen flow at a heating rate of 10 °C/min. Differential scanning calorimetry (DSC) measurements were performed on Netzsch DSC 209 under nitrogen flow at several heating and cooling cycle with heating rate of 10 °C/min and cooling rate of 20°C/min. Ultraviolet–visible absorption spectra was recorded using Perkin-Elmer Lambda 950-PKA UV/VIS while Photoluminescence (PL) spectra was recorded by (Horiba Jobin Yvon) FluoroMax-4 spectrofluorometer, respectively. Cyclic voltammetry (CV) was carried out on the CHI-600D electrochemical workstation using Platimum as working electrode and a Pt wire as counter electrode and Ag/AgCl as reference electrode at a scanning rate of 100 mV/s in nitrogen-saturated 0.1 mol/L n-Bu_4NPF_6 anhydrous acetonitrile and dichloromethane (DCM) solution. PL quantum yields (PLQYs) of the solution and doped films were measured by using an integrating sphere on HAMAMATSU absolute PL quantum yield spectrometer (C11347). Nitrogen bubbling was conducted to eliminate oxygen in diluted solutions while integrating sphere was degassed with nitrogen for doped films measurement. Transient PL at room temperature was measured using Quantaurus-Tau fluorescence lifetime measurement system (C11367-03, Hamamatsu Photonics Co., Japan). Film samples were measured under N_2 atmosphere. All decay was evaluated on TCC900 mode with 340 nm LED excitation source. Transient PL of film samples at various temperatures were investigated under N_2 atmosphere using Quantaurus-Tau fluorescence lifetime measurement system (C11367-03, Hamamatsu Photonics Co., Japan) equipped with Oxford Instruments nitrogen cryostat (Optistat DN).

Quantum Chemical Calculations All of the simulations were performed using the Gaussian 09_B01 program package.[1] More detailed calculation of optimal HF% (OHF), E_{VA} (S_1) and E_{VE} (S_1) are shown in Fig. S7 and Table S2.[2, 3] Intuitive comparison of S_0 and S_1 geometries with minimum RMSD were calculated by VMD software. Theoretical absorption simulation was carried out using Multiwfn software.[4]

Device Fabrication and Characterization Glass substrates pre-coated with a 95-nm-thin layer of indium tin oxide (ITO) with a sheet resistance of 10 Ω per square were thoroughly cleaned in ultrasonic bath of acetone, isopropyl alcohol, detergent, deionized water, and isopropyl alcohol and treated with O_2 plasma for 20 min in sequence. Organic layers were deposited onto the ITO-coated glass substrates by thermal evaporation under high vacuum ($<5\times10^{-4}$ Pa). Cathode, consisting of a 1 nm-thin layer of LiF followed by a 100 nm thin Al layer, was patterned using a shadow mask with an array of 3 mm × 3 mm openings. Deposition rates are 1–2 Å/s for organic materials, 0.1 Å/s for LiF, and 6 Å/s for Al, respectively. Electroluminescence (EL) spectra were recorded by an optical analyzer, Photo Research PR705. The current density and luminance versus driving voltage characteristics were measured by Keithley 2420 and Konica Minolta chromameter CS-200. EQE was calculated from the luminance, current density, and EL spectrum, assuming a Lambertian distribution.

2. Supplemental Tables and Figures

a) Synthesis and Characterization

All compounds were easily purified by column chromatography and train sublimation. Chemical structures of all compounds were fully characterized by NMR (^{1}H and ^{13}C) and atmosphere pressure chemical ionization mass spectra (APCI).

Synthesis of 1,2-bis(4-(3,6-di-*tert*-butyl-9H-carbazol-9-yl)phenyl)ethane-1,2-dione (DC-TC)

1,2-Bis(4-bromophenyl)ethane-1,2-dione (368 mg, 1 mmol), 3,6-di-*tert*-butyl-9H-carbazole (586 mg, 2.1 mmol), and K_2CO_3 (828 mg, 6 mmol) were added into a three neck flask in 50 ml toluene in N_2 atmosphere. After degassing for 15 min, acetic acid palladium (II) (22.4 mg, 0.1 mmol) and tri-*tert*-butylphosphine (0.36 ml, 0.36 mmol) were added. Subsequently, the mixture was stirred and refluxed overnight. After removing the solvent in vacuum, the mixture was partitioned between DCM and water. The combined organic layers were washed with brine, dried over Mg_2SO_4 and concentrated in vacuo. Column chromatography of the residue solid (eluent: DCM/PE=1/3) afforded 703 mg of DC-TC. The yield is over 92%.

^{1}H NMR (500 MHz, $CDCl_3$) δ 8.31 – 8.26 (m, 4H), 8.14 (t, *J* = 1.2 Hz, 4H), 7.83 – 7.79 (m, 4H), 7.52 – 7.46 (m, 8H), 1.51 – 1.43 (m, 36H). ^{13}C NMR (126 MHz, $CDCl_3$) δ 192.91, 144.57, 144.12, 138.22, 131.88, 130.48, 126.04, 124.21, 124.01, 116.51, 109.39, 34.80, 31.94. APCI-MS m/z Calcd for $C_{54}H_{56}N_2O_2$, 765.05; found, 765.7.

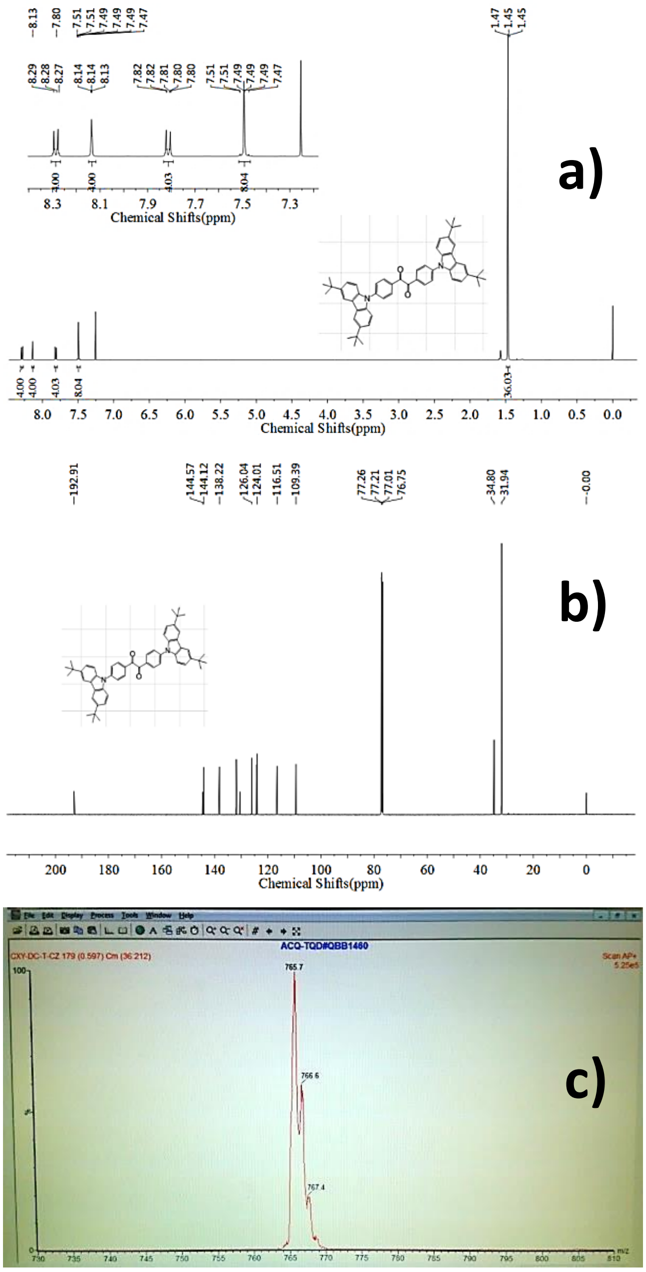

Fig. S1 a) ^{1}H NMR, b) ^{13}C NMR and c) APCI MS spectra of DC-TC.

Synthesis of 1,2-bis(4-(9,9-dimethylacridin-10(9H)-yl)phenyl)ethane-1,2-dione (DC-ACR)

1,2-Bis(4-bromophenyl)ethane-1,2-dione (368 mg, 1 mmol), 9,9-dimethyl-9,10-dihydroacrid- ine (440 mg, 2.1 mmol), and K_2CO_3 (828 mg, 6 mmol) were added into a three neck flask in 50 ml toluene in N_2 atmosphere. After degassing for 15 min, acetic acid palladium(II) (22.4 mg, 0.1 mmol) and tri-*tert*-butylphosphine (0.36 ml, 0.36 mmol) were added. Subsequently, the mixture was stirred and refluxed overnight. After removing the solvent in vacuum, the mixture was partitioned between DCM and water. The combined organic layers were washed with brine, dried over Mg_2SO_4 and concentrated in vacuo. Column chromatography of the residue solid (eluent: DCM/PE=1/2) afforded 530 mg of DC-ACR. The yield is over 85%. ^{1}H NMR (500 MHz, $CDCl_3$) δ 8.31 – 8.19 (m, 4H), 7.57 – 7.51 (m, 4H), 7.48 (dt, J = 11.1, 5.4 Hz, 4H), 7.08 – 6.97 (m, 8H), 6.52 – 6.44 (m, 4H), 1.65 (s, 12H). ^{13}C NMR (126 MHz, $CDCl_3$) δ 192.88, 148.31, 140.24, 132.67, 131.10, 128.99, 126.42, 125.32, 122.01,

115.99, 36.40, 30.52. APCI-MS m/z Calcd for $C_{44}H_{36}N_2O_2$, 624.78; found, 625.4.

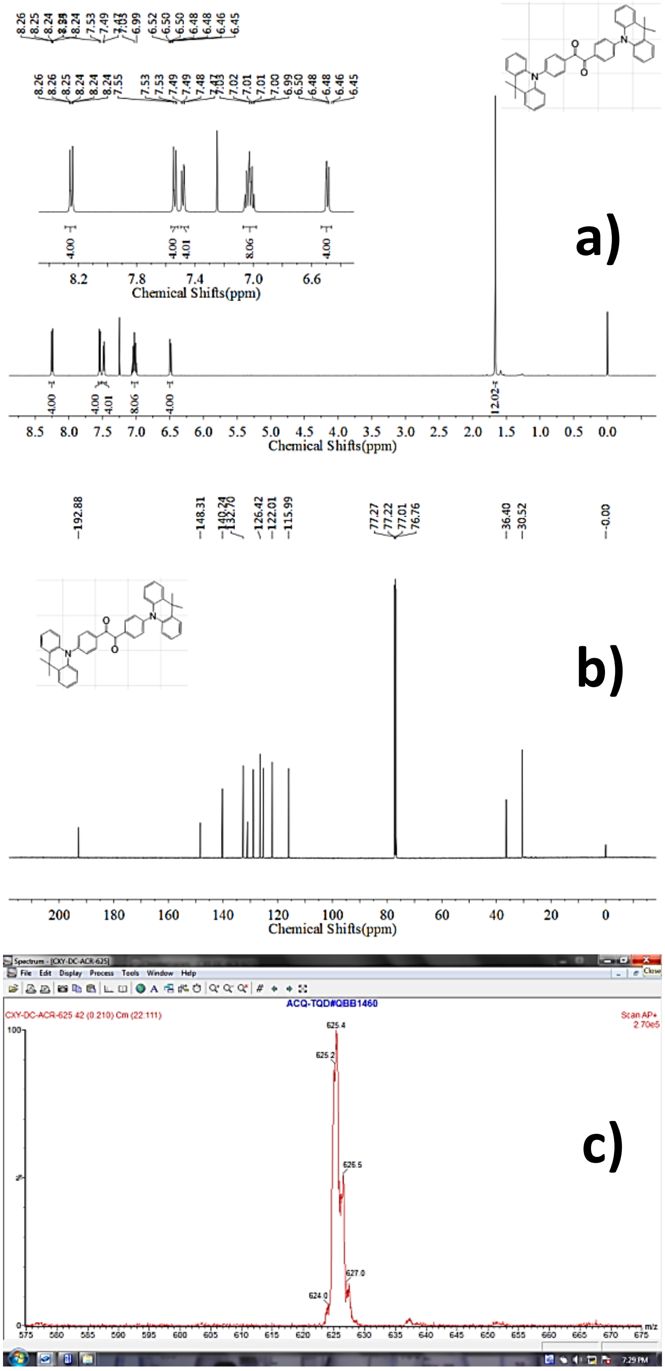

Fig. S2 a) ^{1}H NMR, b) ^{13}C NMR and c) APCI MS spectra of DC-ACR.

Synthesis of 5,6-bis(4-(3,6-di-*tert*-butyl-9H-carbazol-9-yl)phenyl)pyrazine-2,3- dicarbonitrile (PyCN-TC)

1,2-Bis(4-(3,6-di-*tert*-butyl-9H-carbazol-9-yl)phenyl)ethane-1,2-dione (DC-TC) (765 mg, 1 mmol) and diaminomaleonitrile (DAMN) (119 mg, 1.1 mmol) were added into 100 mL AcOH, then heated to 120°C and stirred overnight. Excess AcOH was removed in vacuum and the mixture was washed with water. Column chromatography of the residue solid (eluent: DCM/PE=1/2) afforded 780 mg of PyCN-TC. The yield is over 93%. ^{1}H NMR (500 MHz, $CDCl_3$) δ 8.15 (d, J = 1.6 Hz, 4H), 7.91 – 7.81 (m, 4H), 7.74 – 7.64 (m, 4H), 7.53 – 7.47 (m, 4H), 7.47 – 7.40 (m, 4H), 1.47 (s, 36H). ^{13}C NMR (126 MHz,

$CDCl_3$) δ 154.13, 143.95, 141.33, 138.28, 132.71, 131.45, 129.67, 126.12, 124.00, 116.55, 113.08, 109.24, 34.80, 31.95. APCI-MS m/z Calcd for $C_{58}H_{56}N_6$, 837.13; found, 837.6.

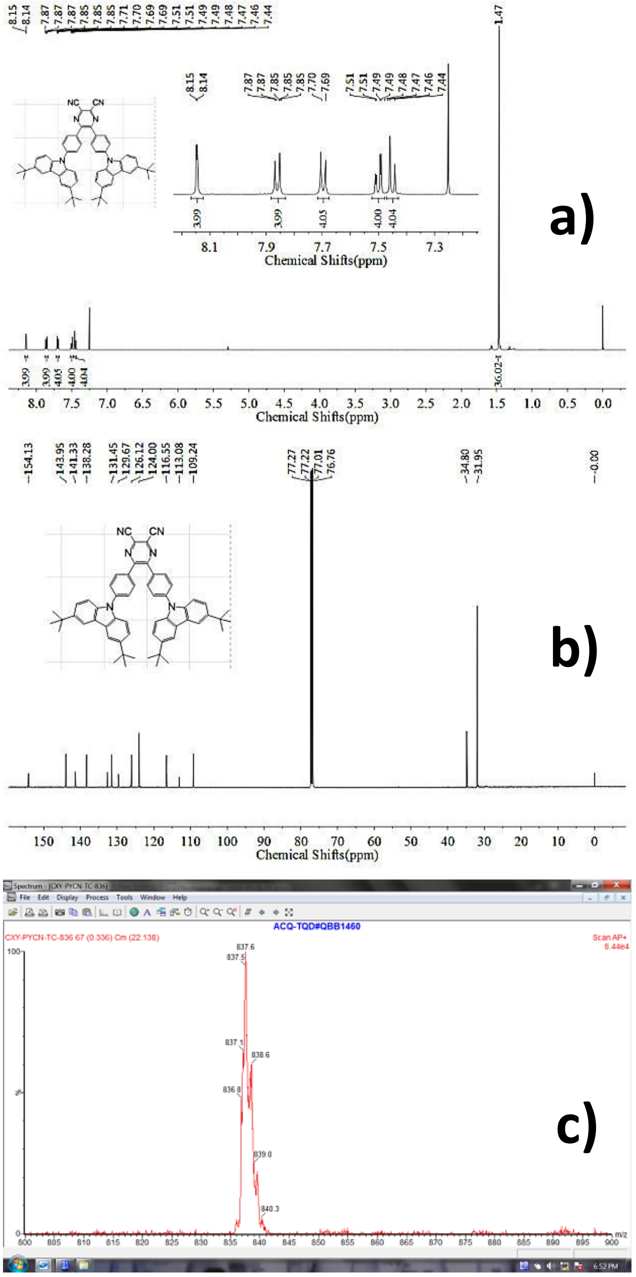

Fig. S3 a) ^{1}H NMR, b) ^{13}C NMR and c) APCI MS spectra of PyCN-TC.

Synthesis of 5,6-bis(4-(9,9-dimethylacridin-10(9H)-yl)phenyl)pyrazine-2,3- dicarbonitrile (PyCN-ACR)

1,2-Bis(4-(9,9-dimethylacridin-10(9H)-yl)phenyl)ethane-1,2-dione (DC-ACR) (625 mg, 1 mmol) and diaminomaleonitrile (DAMN) (119 mg, 1.1 mmol) were added into 100 mL AcOH, then heated to 120°C and stirred overnight. Excess AcOH was removed in vacuum and the mixture was washed with water. Column chromatography of the residue solid (eluent: DCM/PE=1/1) afforded 601 mg of PyCN-ACR. The yield is over 86%.^{1}H NMR (500 MHz, $CDCl_3$) δ 7.90 – 7.80 (m, 4H), 7.45 (ddd, J = 8.9, 5.9, 2.1 Hz, 8H), 6.93 (pd, J = 7.2, 1.6 Hz, 8H), 6.35 (dd, J = 7.8, 1.5 Hz, 4H), 1.66 (s, 12H). ^{13}C NMR (126 MHz, $CDCl_3$) δ 154.77, 144.67, 140.35, 134.21, 132.34, 131.40, 130.84, 130.16, 126.48, 125.28, 121.48,

114.63, 113.05, 36.18, 30.66. APCI-MS m/z Calcd for $C_{48}H_{36}N_6$, 697.5; found, 696.86.

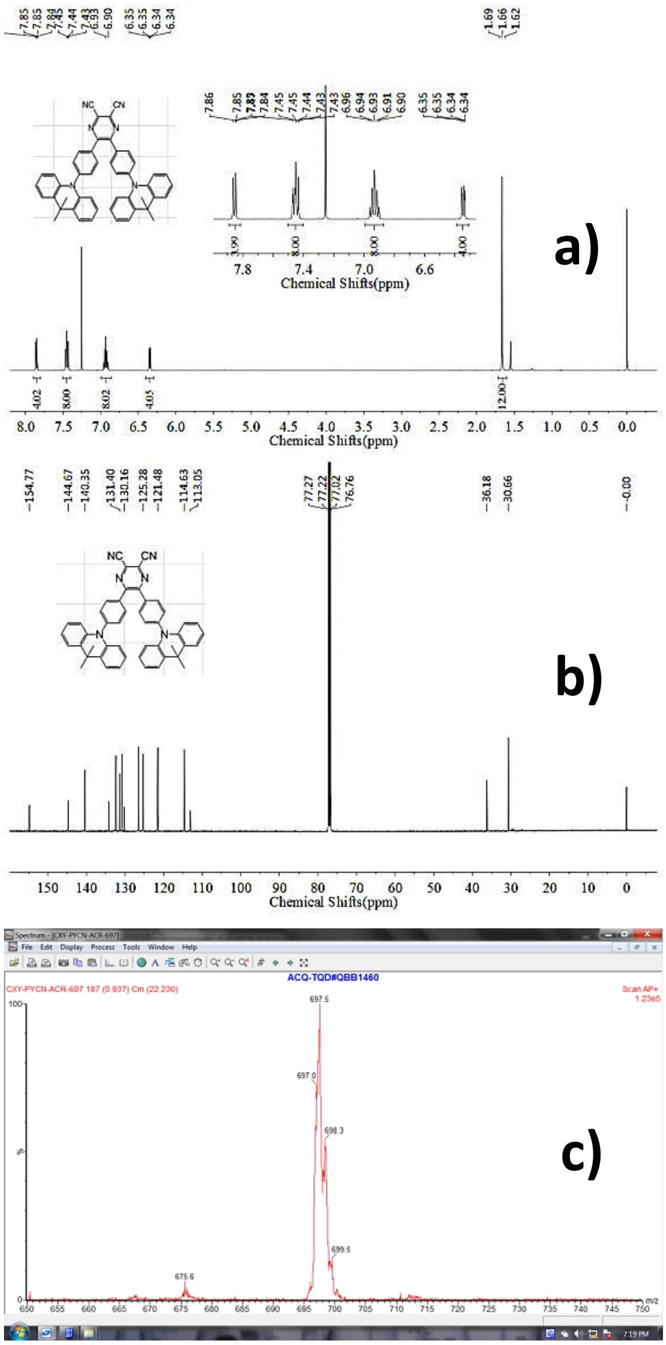

Fig. S4 a) ^{1}H NMR, b) ^{13}C NMR and c) APCI MS spectra of PyCN-ACR.

b) Quantum Chemical Computation

Compound	Excited State	Hole	Particle	Eigenvalue	f
DC-TC	$E_{VA}(S_1)$	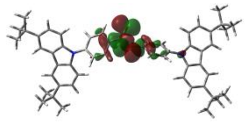	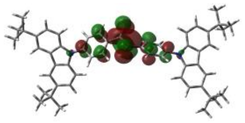	96.1	0.0091
	$E_{VA}(S_2)$	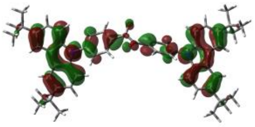	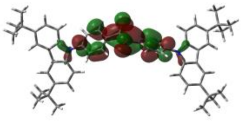	90.3	0.7712
		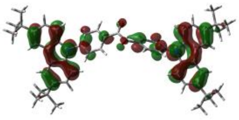	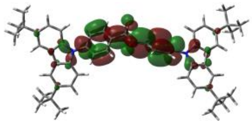	9.2	
	$E_{VE}(S_1)$	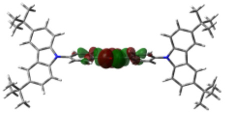	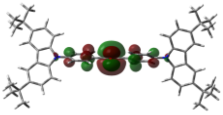	97.8	0.0000
	$E_{VE}(S_2)$	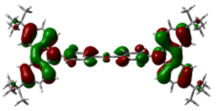	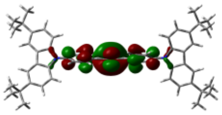	96.9	0.5561
DC-ACR	$E_{VA}(S_1)$	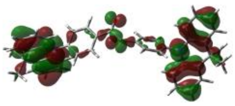	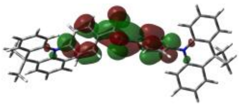	87.8	0.0001
		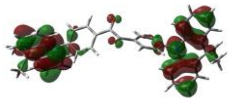	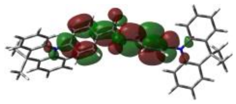	11.2	
	$E_{VE}(S_1)$	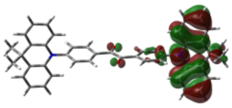	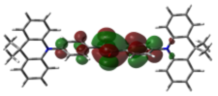	99.8	0.0011

Fig. S5. NTO transition analysis with different eigenvalues of DC-TC and DC-ACR are listed for comparison. Note that the HONTO and LUNTO overlap on central benzil moieties are hardly contributing to increase in oscillator strength given that the n-π* transition character of benzil core leads to small overlap integral. Therefore, very small f was observed in TD-DFT computation.

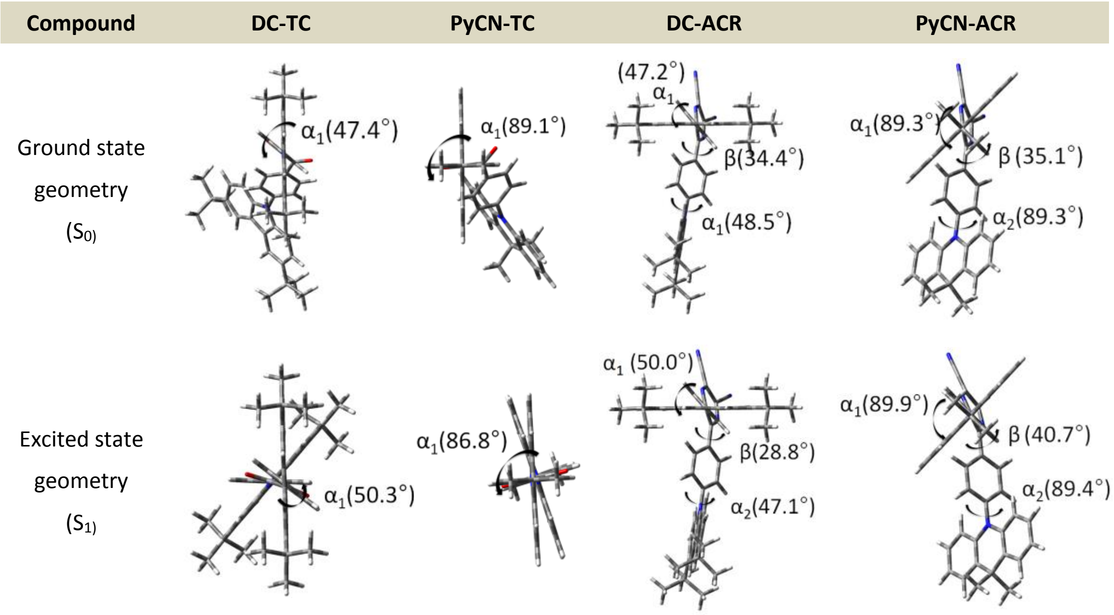

Fig. S6. Side views of DFT-optimized ground state geometries on B3LYP/6-31G* in gas phase and excited state geometries on BMK/6-31G* in toluene using PCM model of the four emitters. Dihedral angles are displayed for intuitive comparison between TC-Ph and ACR-Ph.

Table S1. Calculated transition energies (E_{VA} and E_{VE}), oscillator strengths (f_{VA} and f_{VE}) and configuration interaction description of frontier orbital transitions using optimized HF% functionals, and 6-31G* basis set in toluene based on optimized ground state geometry and excited state geometry, respectively. a) denotes E_{VE} in n-hexane for comparison with E_{VE} in toluene. For comparison, calculated results based on M06-2x/6-31G* are also listed.

Emitters	functional		**S_0 geometry in gas phase**				**S_1 geometry in toluene**			
			E_{VA}(eV) (nm)	CI description	C_j%	f_{VA}	E_{VE}(eV) (nm)	CI description	C_j%	f_{VE}
DC-TC	BMK	S_1	2.9212 (424.5)	H-6-L<br>H-10-L<br>H-8-L	77.6<br>4.1<br>3.5	0.0091	2.4559 (504.9)	H-10-L<br>H-7-L<br>H-6-L	6.2<br>17.0<br>70.2	0.0000
		S_2	3.2310 (383.8)	H-1-L+1<br>H-L	8.6<br>85.0	0.7712	2.8032 (442.4)	H-L+1<br>H-1-L	2.7<br>94.6	0.5561
PyCN-TC	BMK	S_1	2.8493 (435.2)	H-1-L+1<br>H-1-L	3.0<br>78.0	0.3632	2.5474 (486.8)	H-L<br>H-4-L	91.6<br>3.98	0.4677
		S_2	3.0508 (406.4)	H-1-L<br>H-L+1	90.0<br>6.4	0.3774	2.8207 (439.6)	H-1-L	95.7	0.3277
DC-ACR	BMK	S_1	2.7661 (448.2)	H-L<br>H-1-L+1	79.1<br>10.3	0.0001	1.9340 (641.1)	H-L<br>H-L+1	95.3<br>2.0	0.0002
		S_2	2.7793 (446.1)	H-1-L<br>H-L+1	85.8<br>11.7	0.0002	2.0579 (602.5)	H-1-L<br>H-1-L+1	94.0<br>2.0	0.0011
	M06-2x	S_1	2.8525 (434.7)	H-8-L<br>H-10-L<br>H-12-L	56.5<br>11.3<br>16.6	0.0004	2.3702 (523.2)	H-L<br>H-8-L<br>H-10-L<br>H-12-L	3.0<br>23.5<br>16.6<br>48.0	0.0000
		S_2	3.1584 (392.6)	H-1-L<br>H-L+1	75.4<br>19.5	0.0003	2.8326 (437.8)	H-L+1<br>H-1-L	6.7<br>90.8	0.0085
		S_1 a)					2.3778 a) (521.4) a)			0.0014a)
		S_2 a)					2.8497 a) (435.1) a)			0.1399 a)
PyCN-ACR	BMK	S_1	2.5862 (479.4)	H-L<br>H-1-L+1	6.0<br>89.0	0.0000	2.1105 (588)	H-L	95.8	0.0022
		S_2	2.5863 (479.4)	H-1-L<br>H-L+1	89.0<br>6.0	0.0001	2.3857 (519.7)	H-1-L	96.9	0.0025
	M06-2x	S_1	3.0114 (411.7)	H-L<br>H-L+1<br>H-1-L<br>H-1-L+2	4.2<br>8.8<br>78.0<br>3.4	0.0001	2.4780 (500.3)	H-L<br>H-L+2	92.8<br>3.1	0.0005
		S_2	3.0115	H-L	78.0	0.0000	2.8733	H-1-L	93.33.4	0.0000

	(411.7)	H-1-L	4.2	(431.5)	H-1-L+1
		H-1-L+1	8.9		
		H-L+2	3.4		

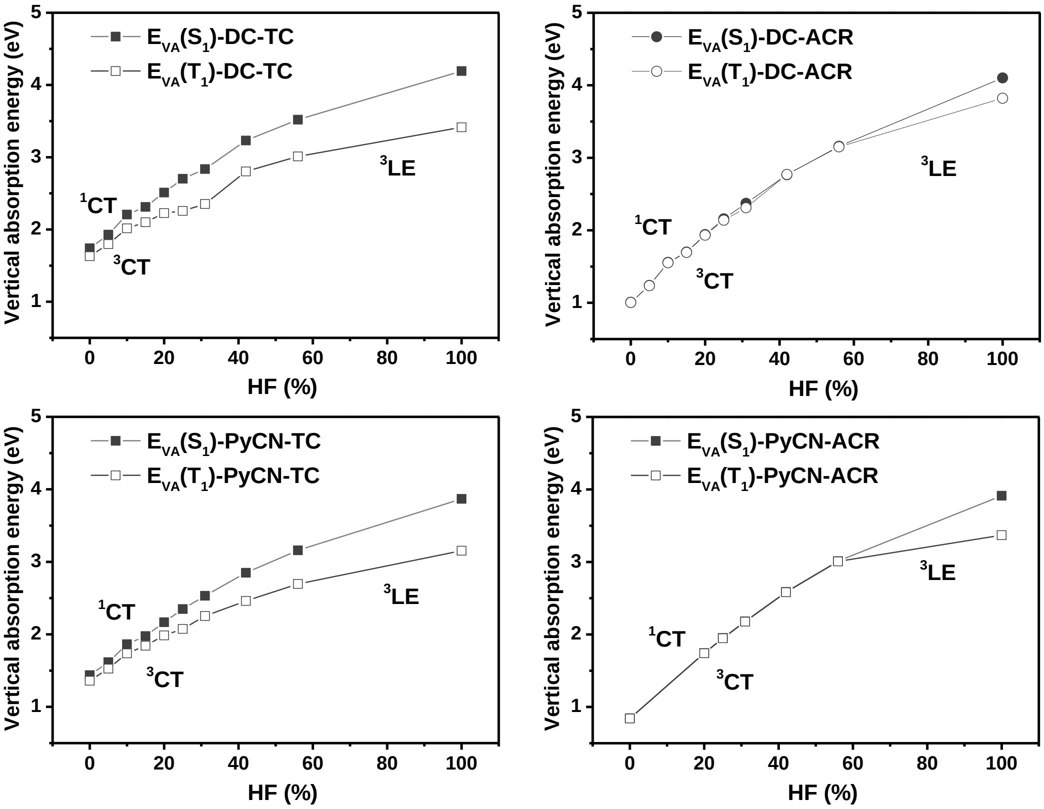

Fig. S7. Dependence of E_{VA} (S_1) and E_{VA} (T_1) on the HF% for DC-TC, DC-ACR, PyCN-TC, and PyCN-ACR in TD-DFT calculation based on different XC functionals.

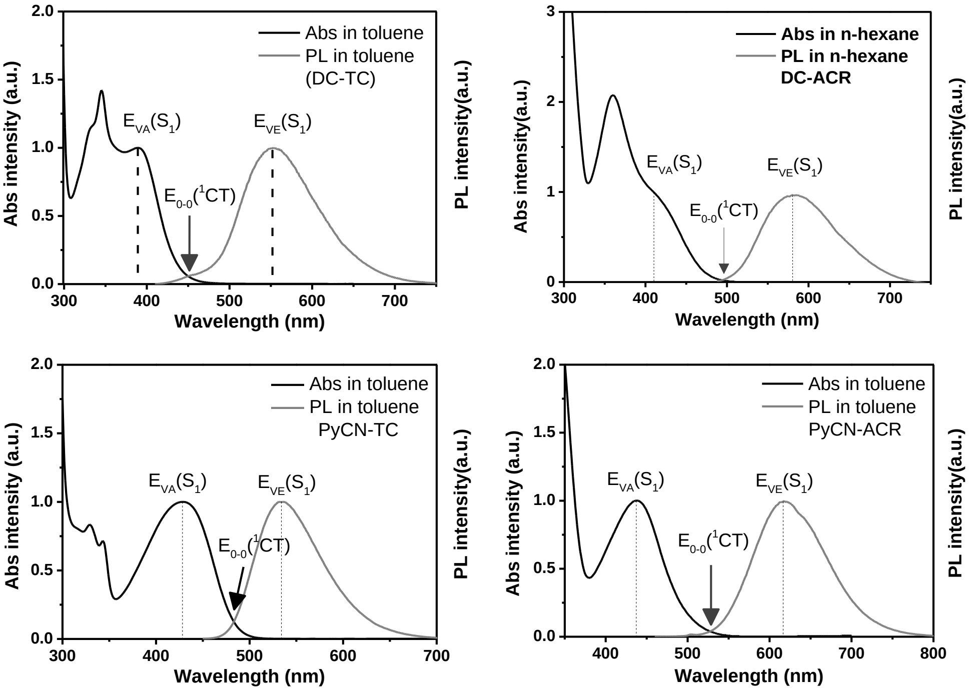

Fig. S8. Absorption and emission spectra of DC-TC, PyCN-TC and PyCN-ACR in toluene and DC-ACR in n-hexane at

300 K.

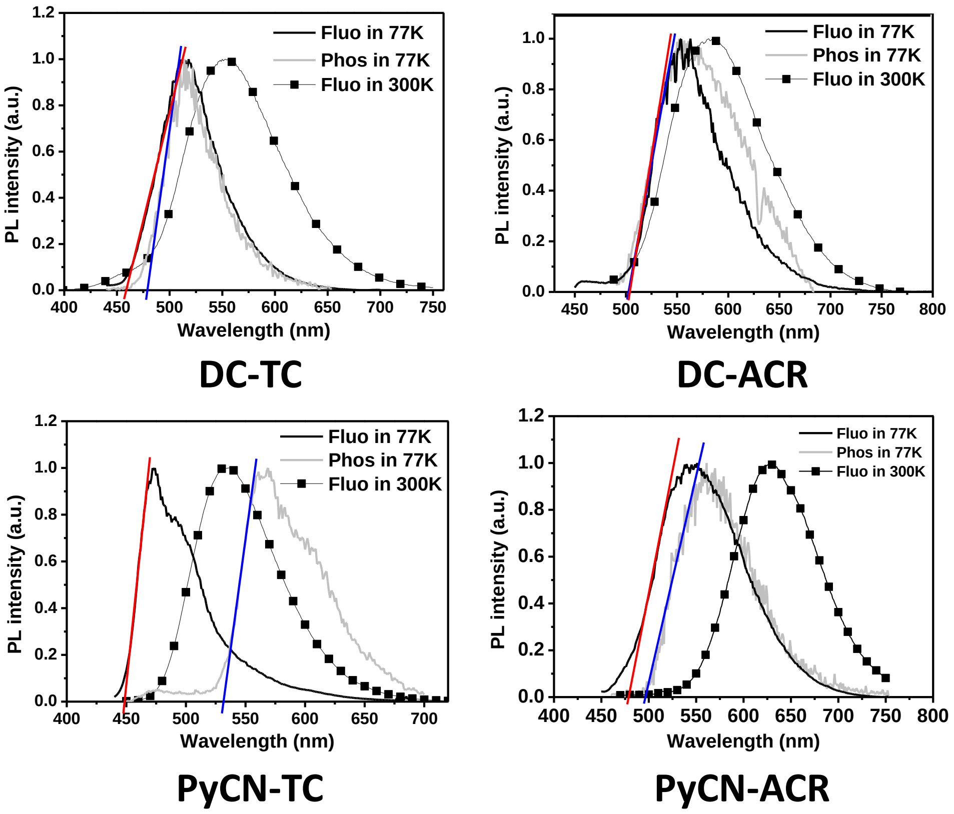

Fig. S9. Phosphorescence emission spectra of DC-TC, PyCN-TC and PyCN-ACR in toluene and DC-ACR in n-hexane at 77 K. Red and blue lines represent fitting of fluorescence and phosphorescence independently for determination of ΔE_{ST}.

Table S2. Fitted optimal HF% (OHF), calculated $E_{0\text{-}0}$ (^{3}LE), $E_{0\text{-}0}$ (^{1}CT) and $E_{0\text{-}0}$ (^{3}CT) of the four compounds in toluene (except DC-ACR in n-Hexane) in PCM model. $E_{0\text{-}0}$ (^{3}LE) = E_{VA} (T_1)/C-0.09 eV, where C is 1.10, 1.18, and 1.30 for BMK, M06-2X and M06-HF results, respectively. $E_{0\text{-}0}$ (^{1}CT) = (E_{VA} (S_1, OHF) + E_{VE} (S_1, OHF))/2. $E_{0\text{-}0}$ (^{3}CT) = $E_{0\text{-}0}$ (S_1)-E_{VA} (S_1, OHF) + E_{VA} (S_1, OHF)/E_{VA} (S_1, BLYP)×E_{VA} (T_1, BLYP). Comparison between experimental and computation data is listed. Experimental data for determination of $\triangle E_{ST}$ is obtained in toluene (DC-ACR in n-hexane) at 77K.

parameter	functional	DC-TC	DC-ACR	PyCN-TC	PyCN-ACR
**$E_{VA}(S_1)$ (eV)**	BLYP(0%)[56]	1.7402	1.0075	1.4382	0.8431
	MPWLYP1M(5%)[7]	1.9289	1.2402	1.6168	
	TPSSH(10%)[8]	2.2071	1.5579	1.8643	
	B3LYP*(15%)[9]	2.3118	1.6997	1.9762	
	B3LYP(20%)[10]	2.5111	1.9403	2.1668	1.7435
	PBE0(25%)[1112]	2.7024	2.1581	2.3493	1.9510
	MPW1B95(31%)[13]	2.8368	2.3726	2.5316	2.1799
	BMK(42%)[14]	3.2310	2.7661	2.8493	2.5862
	M062X(56%)[15]	3.5198	3.1584	3.1590	3.0114
	M06HF(100%)[16]	4.1927	4.1023	3.8683	3.9129
**$E_{VA}(T_1)$ (eV)**	BLYP(0%)	1.6300	1.0046	1.3600	0.8417
	MPWLYP1M(5%)	1.7971	1.2367	1.5266	
	TPSSH(10%)	2.0153	1.5523	1.7374	
	B3LYP*(15%)	2.1005	1.6943	1.8428	
	B3LYP(20%)	2.2263	1.9329	1.9861	1.7410
	PBE0(25%)	2.2568	2.1391	2.0751	1.9473
	MPW1B95(31%)	2.3525	2.3108	2.2530	2.1766
	BMK(42%)	2.8034	2.7706	2.4605	2.5819
	M062X(56%)	3.0110	3.1508	2.6951	2.9822
	M06HF(100%)	3.4146	3.8210	3.1537	3.3697
**$E_{0\text{-}0}(^{3}LE)$ (eV)**	BMK(42%)			2.4605	
	M062X(56%)	3.0110		2.6951	
	M06HF(100%)	3.4146	3.8210	3.1537	3.3697
	Average	2.4992	2.9392	2.2256	2.5921
**Optimal HF%**		42	42	42	42
**$E_{VA}(S_1$, OHF) (eV)**	Cal.	3.23	2.77	2.85	2.59
	Exp.	3.19	3.01	2.90	2.84
**$E_{0\text{-}0}(^{1}CT)$ (eV)**	Cal	2.63	2.41	2.69	2.35
	Exp	2.75	2.48	2.57	2.35
**$E_{0\text{-}0}(^{3}CT)/E_{0\text{-}0}(^{3}LE)$ (eV)**	Cal	2.42/2.50	2.40/2.94	2.54/2.23	2.34/2.59
	Exp	2.61	2.47	2.21	2.45
**$\triangle E_{ST}$(eV)**	Cal	0.21	0.01	0.46	0.01
	Exp	0.12	0.01	0.43	0.08

c) Photoluminescence Measurements

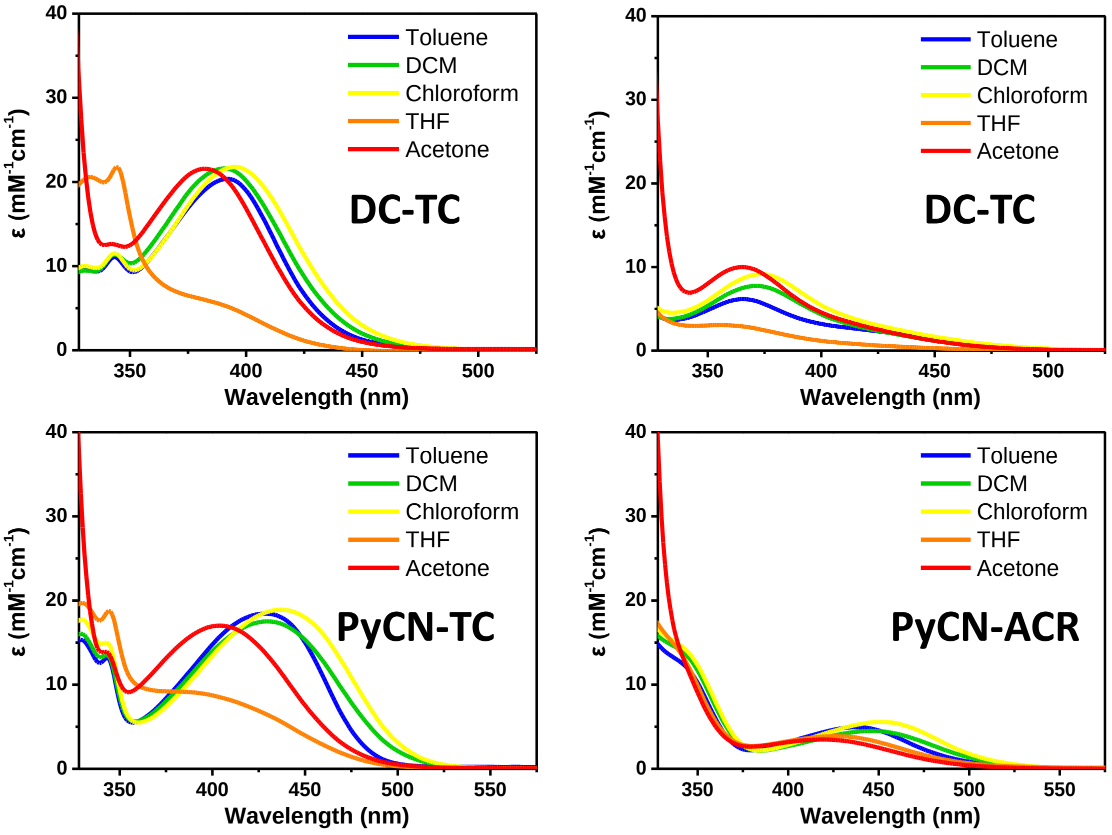

Fig. S10. Solvatochromism absorption spectra of the investigated molecules measured in different solvents (0.05 mM). Molar extinction coefficient and CT absorption peaks presents slight change when changing the solvents.

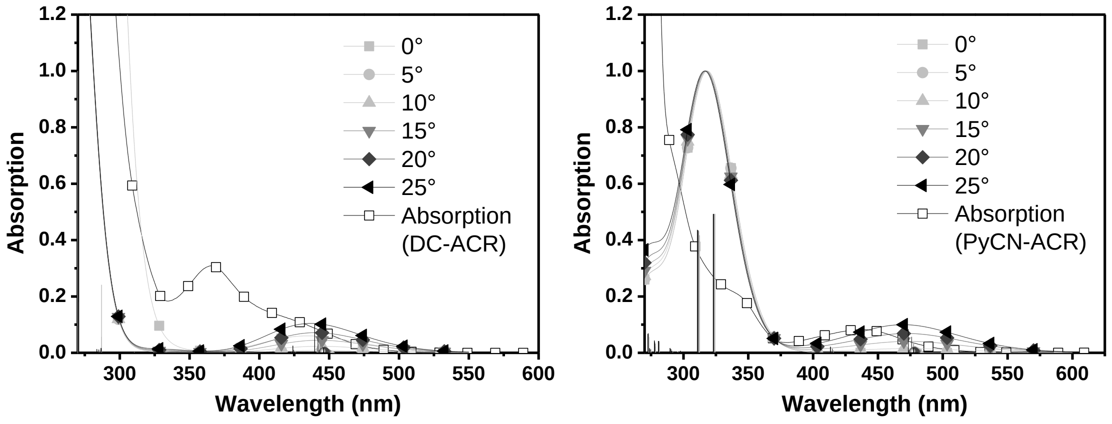

Fig. S11. Theoretical absorption spectra of DC-ACR (left) and PyCN-ACR (right) simulated based on OHF% functionals according to different twisting angles (α) by Multiwfn with half-bandwidth of 0.6 eV. When the twisting angle enlarges from 0 to 25°, the calculated oscillator strength of the longest ICT absorption also increases, explaining the contradiction between TD-DFT (f is close to zero) and experiment (molar extinction coefficient falls into 5-10 mM^{-1}cm^{-1}).

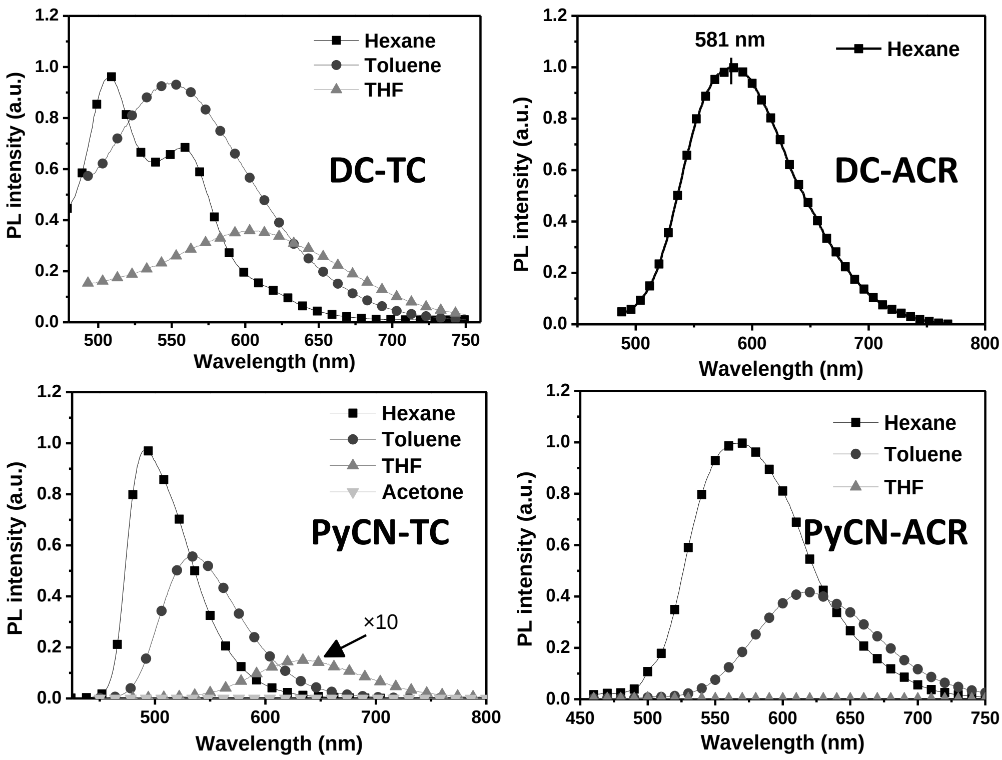

Fig. S12. Solvatochromism PL spectra of the investigated molecules measured in different solvents (0.05 mM).

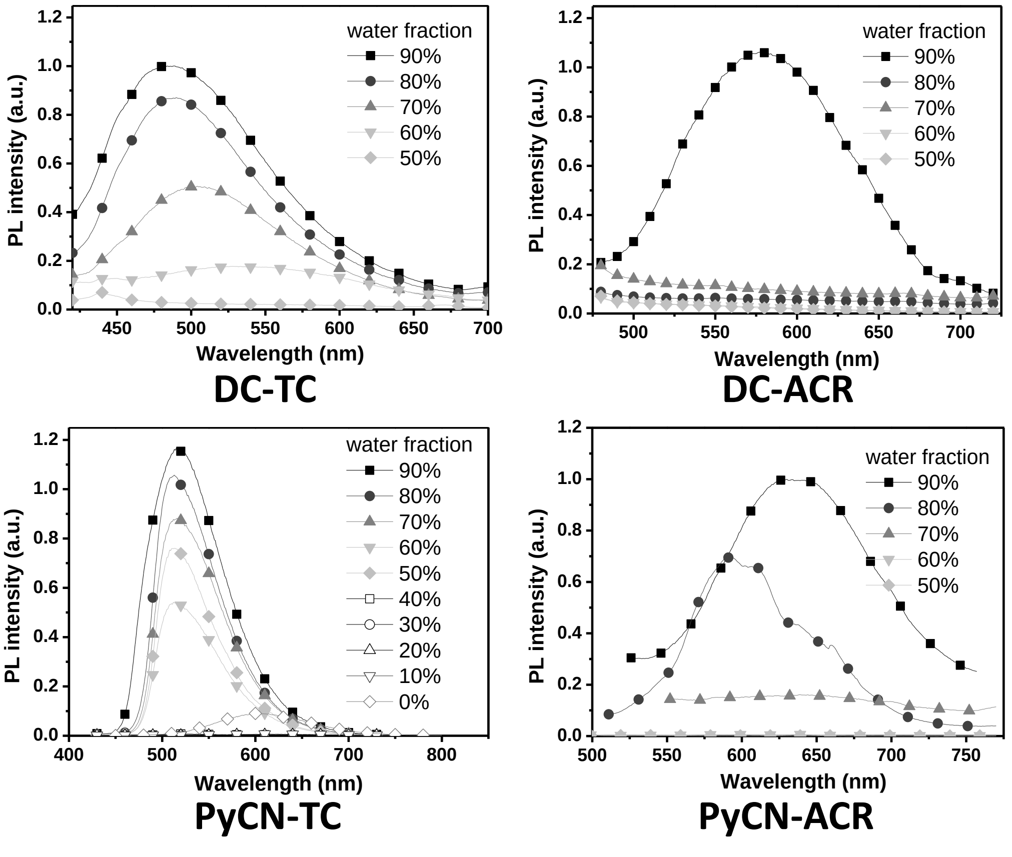

Fig. S13. PL spectra of all compounds in THF/water mixtures with different water fractions.

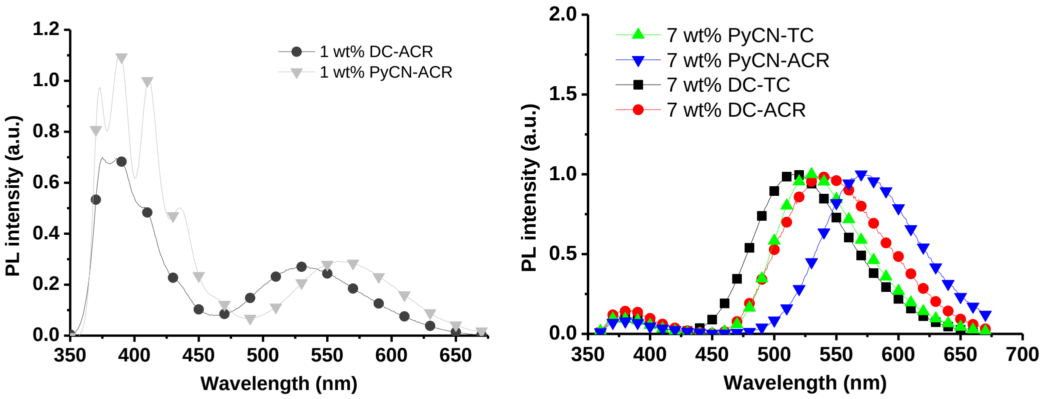

Fig. S14. PL spectra of the investigated molecules doped into CBP films (1 wt% for DC-ACR and PyCN-ACR) and 7 wt% investigated molecules doped into CBP films measured at room temperature. Incomplete energy transfer from host to guest was noted for the left ones while more complete energy transfer was observed for the right ones. To eliminate inaccuracy in measuring PLQY of doped films, 7 wt% doped films were employed to research into optic-physical properties of four compounds. The concentrations of all doped films were controlled at 7 wt% for parallel comparison.

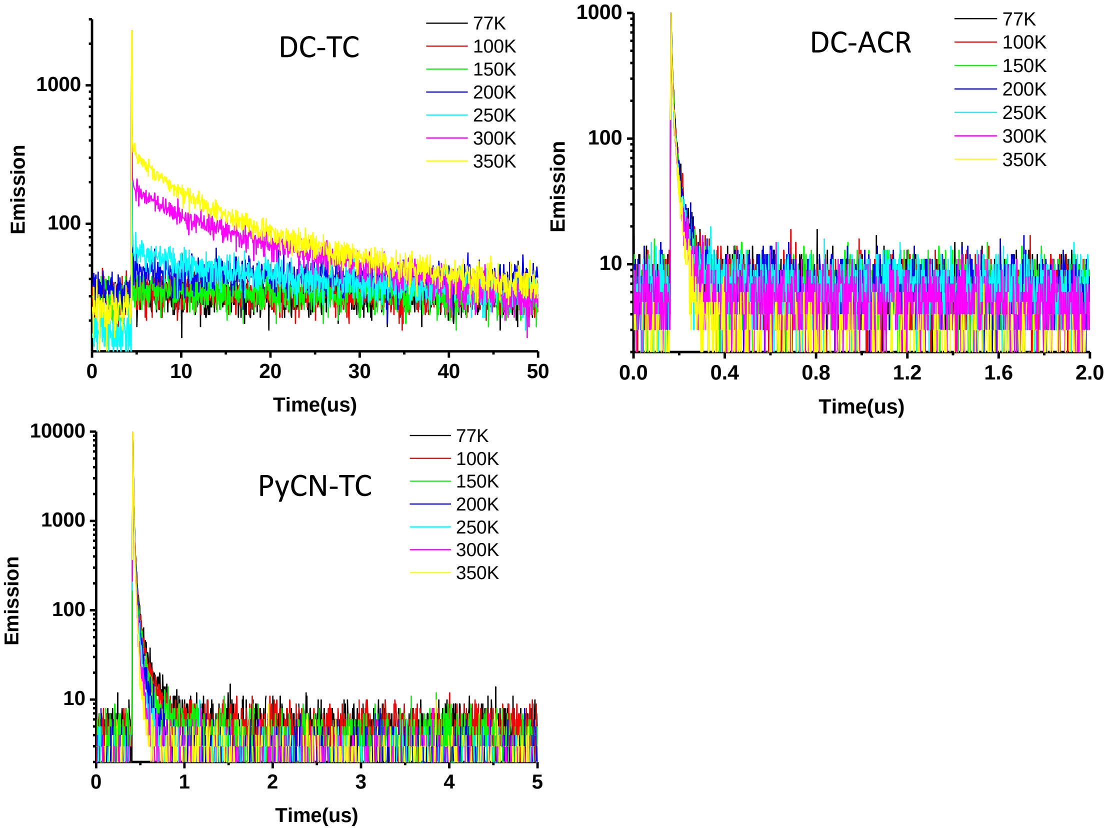

Fig. S15-17. Transient PL decay curves for 5 wt% DC-TC, 5 wt% PyCN-TC and 1 wt% DC-ACR doped into CBP and measured from 77K to 350K.

Table S3. Temperature-dependent transient decay data fit by multi-expotential equation. a) An average lifetime calculated by $\tau_{av} = \Sigma A_i\tau_i^2/\Sigma A_i\tau_i$, where A_i is the pre-exponential for lifetime τ_i. b) Individual component ratio for prompt (n_1) and delayed (n_2) fluorescence.

Name	CHI	EM	<τ>	τ_1 [a)]	τ_2 [a)]	n_1 [b)]	n_2 [b)]
5% DC-TC in CBP at 77K	1.04274	518	0.01830	0.016827	0.104549	0.983131	0.016869
5% DC-TC in CBP at 100K	1.03235		0.01702	0.016142	0.054085	0.976797	0.023203
5% DC-TC in CBP at 200K	1.06043		0.02202	0.011684	0.084961	0.858856	0.141144
5% DC-TC in CBP at 250K	1.09027		21.8018	0.01512	36.7048	0.406189	0.593811
5% DC-TC in CBP at 300K	0.98887		18.7484	0.012645	24.92422	0.247922	0.752078
5% DC-TC in CBP at 350K	1.08776		10.1759	0.016876	12.47564	0.184588	0.815412
1% DC-ACR in CBP at 77K	1.16818	532	0.01843	0.00293	0.023894	0.260393	0.739607
1% DC-ACR in CBP at 100K	1.21211		0.02106	0.00356	0.029958	0.337131	0.66286
1% DC-ACR in CBP at 150K	1.14378		0.01756	0.003315	0.024415	0.324637	0.675363
1% DC-ACR in CBP at 200K	1.16405		0.01724	0.00065	0.018674	0.079545	0.920455
1% DC-ACR in CBP at 250K	1.26852		0.01680	0.002159	0.022775	0.289755	0.710245
1% DC-ACR in CBP at 300K	1.11164		0.01466	0.00136	0.018712	0.233333	0.76666
1% DC-ACR in CBP at 350K	0.66866		0.01406	0.00253	0.019642	0.326201	0.673799
10% PyCN-TC in CBP at 77K	1.16175	534	0.01901	0.00482	0.054508	0.714277	0.285723
10% PyCN-TC in CBP at 100K	1.0563		0.015331	0.00459	0.041152	0.706378	0.293622
10% PyCN-TC in CBP at 150K	1.03518		0.01333	0.00442	0.032621	0.683901	0.316099
10% PyCN-TC in CBP at 200K	0.73209		0.011305	0.00465	0.024476	0.664476	0.335524
10% PyCN-TC in CBP at 250K	0.61067		0.011395	0.00206	0.016559	0.356097	0.643903
10% PyCN-TC in CBP at 300K	0.673177		0.0091	0.005035	0.018994	0.7111	0.2889
10% PyCN-TC in CBP at 350K	0.613016		0.00799	0.00478	0.018389	0.764325	0.235675
1% PyCN-ACR in CBP at 77K	1.82021	560	0.401943	0.041917	3.75855	0.90313124	0.09686
1% PyCN-ACR in CBP at 100K	1.48449		0.82064	0.036833	4.912250	0.83923251	0.160767
1% PyCN-ACR in CBP at 150K	1.49144		1.93353	0.038341	7.368033	0.7414362	0.258563
1% PyCN-ACR in CBP at 200K	1.29398		4.59007	0.02954	12.789179	0.6425809	0.357419
1% PyCN-ACR in CBP at 250K	1.89758		11.6199	0.039213	20.07840	0.4220031	0.577996
1% PyCN-ACR in CBP at 300K	2.12033		7.95577	0.039823	13.13389	0.3954424	0.604557
1% PyCN-ACR in CBP at 350K	1.7374		3.90469	0.03689	6.9191251	0.4378650	0.562134

d) Device Fabrication and Measurements

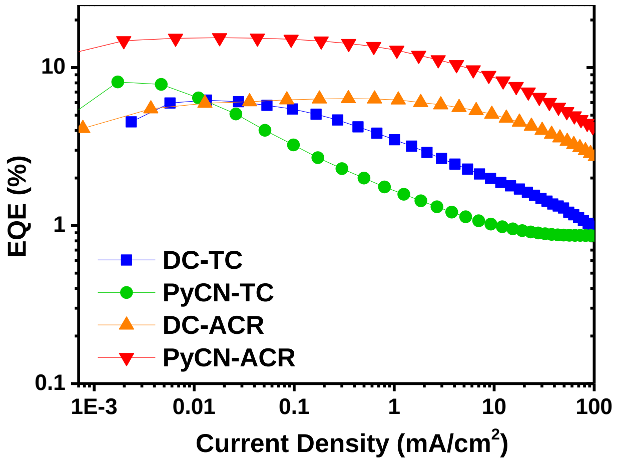

Fig. S18. EQE-current density curves of the OLED devices based on the four investigated molecules in a structure of ITO/ HATCN (5 nm)/ TAPC (20 nm)/ CBP: x wt% emitter (35 nm)/ TmPyPB (55 nm)/ LiF (1 nm)/ Al.

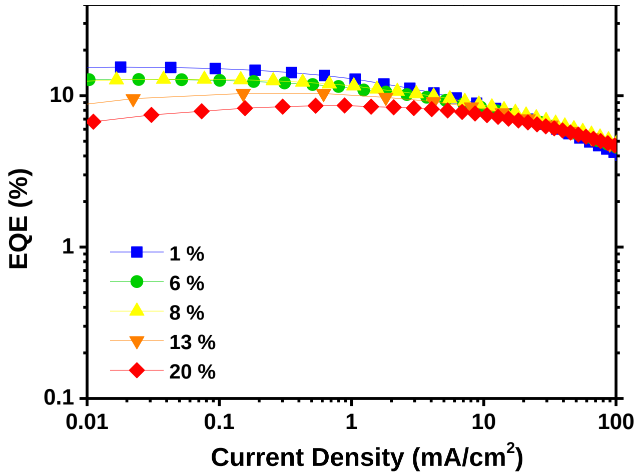

Fig. S19. EQE-current density curves of the OLED devices based on PyCN-ACR at various doping concentrations in a structure of ITO/ HATCN (5 nm)/ TAPC (20 nm)/ CBP: x wt% PyCN-ACR (35 nm)/ TmPyPB (55 nm)/ LiF (1 nm)/ Al.

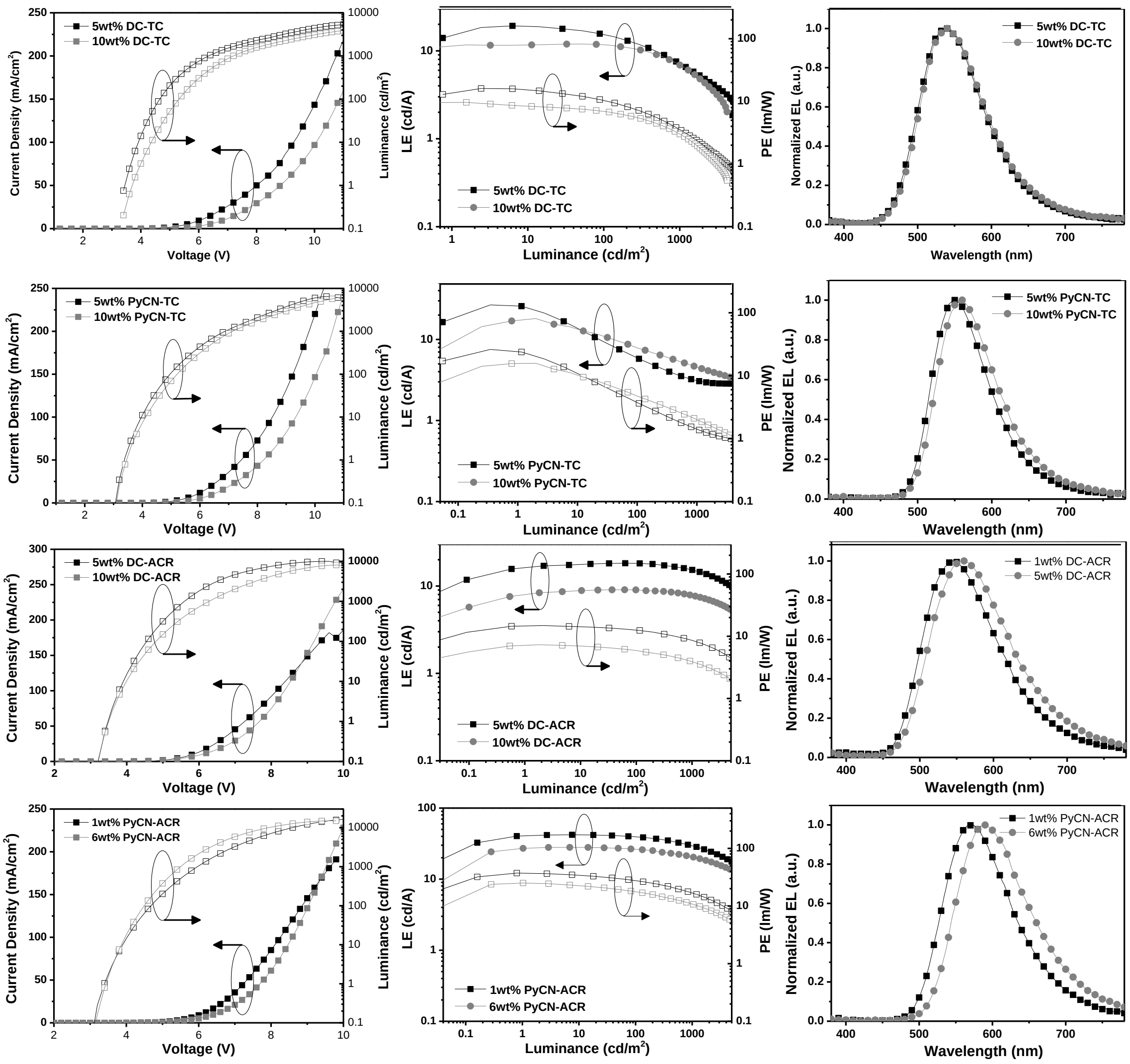

Fig. S20. Concentration quenching effect on the investigated molecules based on different doping concentrations in CBP. Current density-voltage-luminance (J-V-L) and luminance efficiency - luminance - power efficiency (LE-L-PE) and normalized EL spectra are shown. With higher doping concentration, the preference trapping effect of the guests can be enhanced and lower current density was observed because of poorer transporting nature of the guest molecules than CBP. Further evidence from comparing current density-voltage (J-V) curves of different doping concentration of the guests in EML can assist such a deduction. As shown, higher current density can be observed for lower doping concentration of the guest at the same voltage. Interestingly, the degrees of efficiency roll-off are quite close with the devices of different guest doping concentrations. For emitters with large k_{RISC}, efficiency roll-off turns out to be small for all devices, implying that in such a device structure with relatively low doping concentration of the guest emitter, the efficiency roll-off depends mostly on the character of the emitter.

Table S4. Summary of the OLED performance in a device structure of ITO/ HATCN (5 nm)/ TAPC (20 nm)/ CBP: x wt% emitter (35 nm)/ TmPyPB (55 nm)/LiF (1 nm)/ Al.

Device	V_{on}[a] (V)	EQE_{max} (%)	at 100 cd m-2 V (V)	CE (cd A-1)	PE (lm W-1)	CIE (x,y)	EQE (%)	at 1000 cd m-2 V (V)	CE (cd A-1)	PE (lm W-1)	CIE (x,y)	EQE (%)
5 wt% DC-TC	3.4	6.2	4.7	15.0	10.0	(0.349, 0.555)	4.9	6.4	7.0	3.5	(0.348, 0.550)	2.3
10 wt% DC-TC	3.7	3.9	5.3	11.7	7.3	(0.370, 0.556)	3.8	7.2	6.9	3.0	(0.373, 0.553)	2.3
1 wt% DC-ACR	3.5	6.3	4.6	17.9	12.2	(0.386, 0.544)	6.3	5.9	14.6	7.8	(0.386, 0.539)	5.1
5 wt% DC-ACR	3.5	3.4	4.8	9.0	5.9	(0.423, 0.532)	3.3	6.3	7.7	3.9	(0.424, 0.531)	2.9
5 wt% PyCN-TC	3.4	8.1	5.0	5.8	3.6	(0.396, 0.547)	1.8	6.9	3.1	1.4	(0.380, 0.581)	0.9
10 wt% PyCN-TC	3.4	5.8	5.2	7.9	4.8	(0.430, 0.551)	2.5	7.2	4.5	2.0	(0.414, 0.561)	1.4
1 wt% PyCN-ACR	3.4	15.6	4.5	39.4	37.5	(0.464, 0.518)	14.6	5.5	29.5	16.9	(0.464, 0.515)	11.1
6 wt% PyCN-ACR	3.4	12.7	4.7	26.3	17.5	(0.520, 0.474)	12.0	6.0	20.4	10.7	(0.520, 0.474)	9.3

[a]at the luminance of 1 cd/m^2.

Table S5. Summary of the optimal OLED performance in a device structure of ITO/ HATCN (5 nm)/TAPC (20 nm)/ CBP: x wt% emitter (35 nm)/ TmPyPB (55 nm)/ LiF (1 nm)/ Al.

EML	Maximum efficiency			at 100 cd/m^2				at 1000 cd/m^2			
	V_{on}[a] (V)	CE_{max} (cdA-1)	EQE_{max} (%)	V (V)	CE (cd A-1)	CIE (x,y)	EQE (%)	V (V)	CE (cd A-1)	CIE (x,y)	EQE (%)
5 wt% DC-TC	3.4	19.2	6.2	4.7	15.0	(0.349, 0.555)	4.9	6.4	7.0	(0.348, 0.550)	2.3
1 wt% DC-ACR	3.5	18.1	6.3	4.6	17.9	(0.386, 0.544)	6.3	5.9	14.6	(0.386, 0.539)	5.1
5 wt% PyCN-TC	3.4	26.7	8.1	5.0	5.8	(0.396, 0.547)	1.8	6.9	3.1	(0.380, 0.581)	0.9
1 wt% PyCN-ACR	3.4	42.1	15.6	4.5	39.4	(0.464, 0.518)	14.6	5.5	29.5	(0.464, 0.515)	11.1

[a]at the luminance of 1 cd/m^2.

Table S6. Summary of the OLED performance in a device structure of ITO/HATCN (5 nm)/TAPC (20 nm)/ CBP: x wt% PyCN-ACR (35 nm)/ TmPyPB (55 nm)/ LiF (1 nm)/ Al. x varies from 1 to 20.

Device	Maximum Efficiency			at 100 cd m^{-2}				at 1000 cd m^{-2}			
	V_{on}[a] (V)	CE (cdA^{-1})	EQE (%)	V (V)	CE (cd A^{-1})	CIE (x,y)	EQE (%)	V (V)	CE (cd A^{-1})	CIE (x,y)	EQE (%)
1 wt% PyCN-ACR	3.4	42.1	15.6	4.5	39.4	(0.464, 0.518)	14.6	5.5	29.5	(0.464, 0.515)	11.1
6 wt% PyCN-ACR	3.4	28.0	12.7	4.7	26.3	(0.520, 0.474)	12.0	6.0	20.4	(0.520, 0.474)	9.3
8 wt% PyCN-ACR	3.3	27.4	12.9	4.6	23.3	(0.532, 0.463)	12.4	5.9	20.8	(0.528, 0.467)	9.9
13 wt% PyCN-ACR	3.3	19.1	10.3	4.5	19.1	(0.559, 0.438)	10.3	5.7	16.2	(0.554, 0.443)	8.8
20 wt% PyCN-ACR	3.2	14.1	8.6	4.3	14.1	(0.578, 0.420)	8.6	5.8	12.6	(0.572, 0.426)	7.7

[a]at the luminance of 1 cd m^{-2}.

e) TTA and STA roll-off model simulation

The model is fitting according to Ref.[17] When STA and TTA processes are taken into account, the exciton dynamic processes concerning singlet and triplet exciton population can be expressed as[18, 19]:

$$dN_S/dt = -(k_r^S + k_{ISC})N_S + k_{RISC}N_T - k_{ST}N_SN_T + \alpha k_{TT}N_T^2 + J/4de$$

$$dN_T/dt = k_{ISC}N_S - (k_{RISC} + k_{nr}^T)N_T - (1 + \alpha)k_{TT}N_T^2 + 3J/4de$$

Given that d, e and α denote thickness of EML, electron charge and singlet exciton production ratio of TTA, respectively. For TADF-OLED, α is approximated by 0.25 by spin statics. When steady-state current is achieved, i.e. ($dN_S/dt = 0$, $dN_T/dt=0$), the modeled EQE-J curve can be fitted by:

$$\eta_{EQE}(J) = \eta_0 N_S(t = \infty, J)/N_{S0}$$

Through fitting experimental EQE-J curve, k_{ST} and k_{TT} data can be obtained.

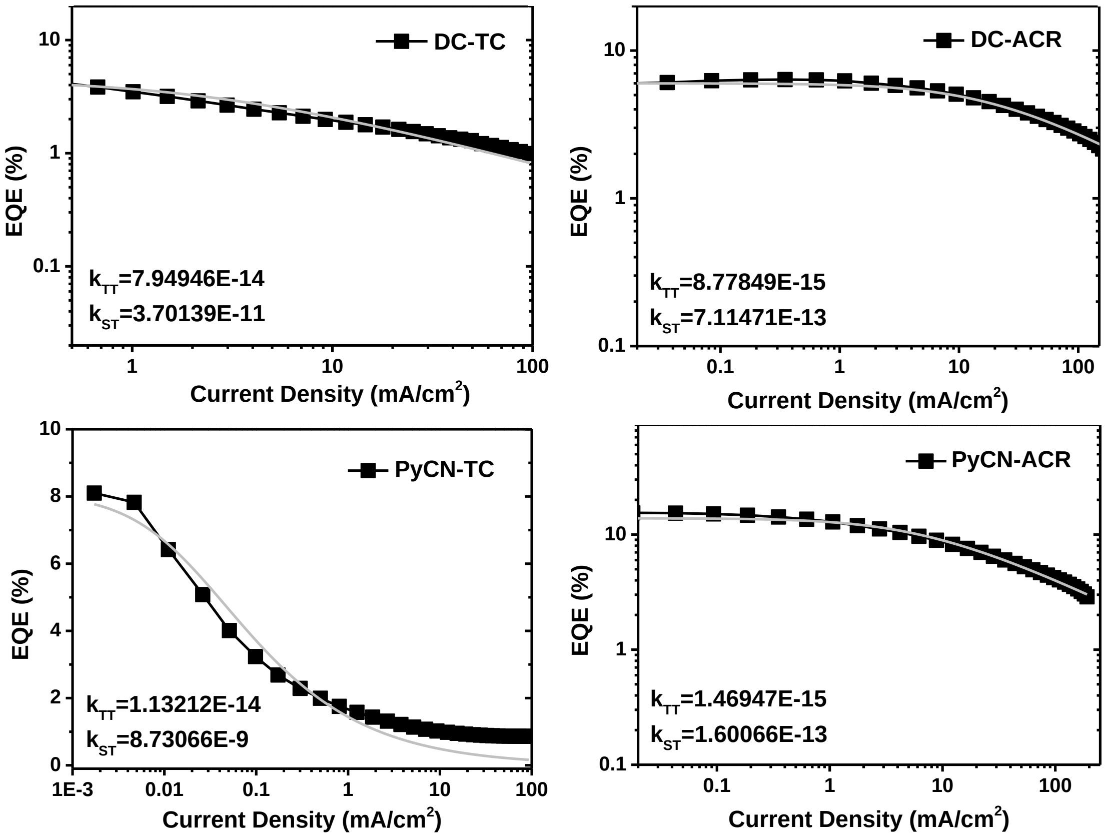

Fig. S21. EQE-J curve and the lines correspond to the calculated EQE based on the model described in the text. The grey fitting line represents the calculated EQE taking TTA and STA processes into account.

f) Thermal Properties

TGA (thermal gravimetric analysis) and differential scanning calorimetry (DSC) was performed to evaluate thermal properties of all compounds. High thermal decomposition temperatures (T_d, corresponding to 5% weight loss) above 400 °C were observed, indicating good thermal stability. Identical raising tendency was observed between T_d and molecular weight. DSC scans on compounds DC-TC and DC-ACR revealed glass transition temperatures (T_g) of 86°C and 98 °C, which was not observed for PyCN-TC and PyCN-ACR. Amorphous and tough films of PyCN compounds can be anticipated due to increased molecular rigidity when rotation free DC- is fixed with PyCN-. Neither melting nor crystallization peak was observed during the heating nor cooling cycles, indicating potential morphology stability in film state for all compounds.

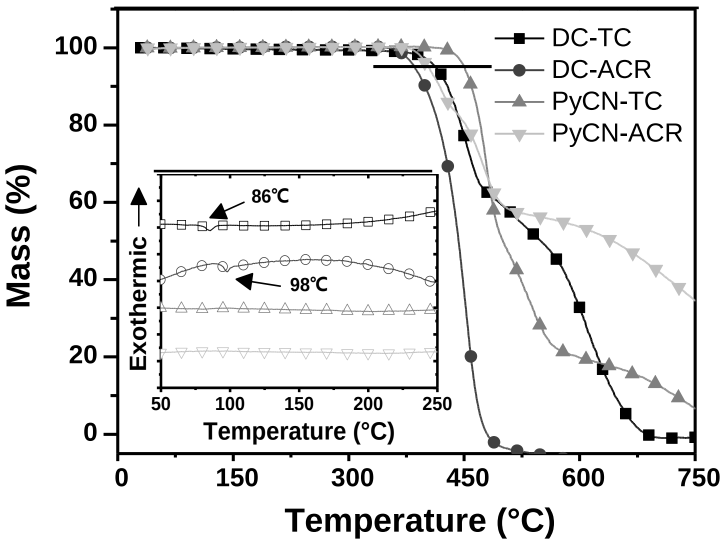

Fig. S22. TGA thermo-grams of all compounds recorded at a heating rate of 20 °C min^{-1}. DSC measurements of all compounds recorded at a heating rate of 10 °C min^{-1} under a flowing nitrogen atmosphere.

g) Cyclic Voltammetry

To understand energy diagrams of all investigated compounds, cyclic voltammetry was carried out using n-Bu_4NPF_6 as supporting electrolyte and referenced to Ag/AgCl electrode. Solid state ionization potential (IP) and electron affinity (EA) are approximated by onset potential of redox peak which may be a theoretically proper way to depict initial injection of holes and electrons to the HOMO and LUMO. Formal potential of ferrocenium/ferrocene (Fc^+/Fc) redox couple (4.8 eV below vacuum level) was evaluated to calibrate IP and EA for investigated molecules.[20] Reversible oxidation wave was observed for DC-TC and PyCN-TC while quasi-reversible oxidation wave was observed for DC-ACR and PyCN-ACR. Blocking of chemical active site by *tert*-butyl unit prevent irreversible oxidations via radical coupling reactions at 3,6- position of carbazole.[21] In contrast, DC-ACR and PyCN-ACR show quasi-reversible oxidation waves that correspond to instability of radical cations on ACR moieties.[22] Neither the reduction potential of DC-TC nor DC-ACR is experimentally accessible. As DC- was chemically modified into PyCN-, reduction scan was noted stably reversible due to chemical stability during the p- and n-doping processes. From these redox potentials, IP (IP(eV)=-e($E_{ox,onset}$+4.34)) of 5.56 eV was estimated for DC-TC and PyCN-TC considering oxidation onset potential at 1.22 V. In comparison, IP of around 5.33 eV of DC-ACR and PyCN-ACR are smaller than that of DC-TC and PyCN-TC, suggesting stronger electron-donating strength of ACR moiety relative to TC. For detectable EA (EA(eV)=-e($E_{red,onset}$ +4.34)) of PyCN- acceptor, onset reduction potential are around 1.02 V, giving EA of around 3.32 eV for PyCN-TC and 3.18 eV for PyCN-ACR.

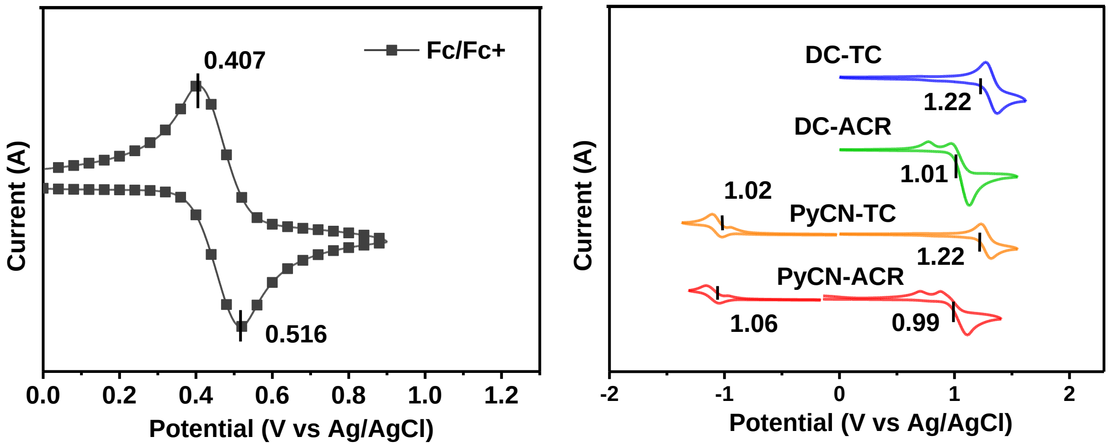

Fig. S23. Reversible redox potential of ferrocene measured in CH_2Cl_2/CH_3CN (4/1) and cyclic voltammograms of all compounds in 0.1 M n-Bu_4NPF_6 in CH_2Cl_2/acetonitrile (4/1) solution.

Evaluation of electrochemical gap was also conducted based on differences between the onsets of p- and n-doping. The onset potentials showing in Fig. 3b are calculating to afford the resulting $E_{g,EC,onset}$ = e(E^{ox}_{onset} - E^{red}_{onset}). However, unexpected $E_{g,EC,onset} < E_{g,opt}$ (optical gap estimated from absorption onset in toluene) was found and the alike phenomenon have also been reported by Andersson, etc when studying electrochemical bandgaps of substituted polythiophenes. The known $E_{g,EC,onset}$ approximates so called fundamental gap (IP-EA) while $E_{g,opt}$ represents energy of lowest electronic transition via absorption of single photon. In the optical excited state,

electrostatically bound hole and electron are formed and difference between fundamental gap and optical gap defines the exciton binding energy.[23] For compounds PyCN-TC and PyCN-ACR, strong intramolecular charge transfer state leads to formation of weakly bound excitons.[24] The spatially separated frontier orbital distribution on HOMO and LUMO visualized from DFT simulation endows small exciton binding energy, which may results in proximation of $E_{g,EC,onset}$ and $E_{g,opt}$ (2.24 and 2.30 eV for PyCN-TC and 2.15 and 2.16 eV for PyCN-ACR). Furthermore, the measured lowest optical transition onset may not correspond to the lowest transition orbital. As shown in Table 1, lack of overlap on HOMO and LUMO for PyCN-ACR induces negligible oscillator strength as small as 0.0001, which may not be easily recognized for onset of absorption and overestimation of $E_{g,opt}$ can be conducted. Last but not least, cyclic voltammetry was carried out in solution and solvent or supporting electrolyte effects should account for the error margins larger than ±0.1 eV in optimal cases.[20]

3. Reference

1 M. J. Frisch, G. W. Trucks, H. B. Schlegel, G. E. Scuseria, M. A. Robb, J. R. Cheeseman, G. Scalmani, V. Barone, B. Mennucci, G. A. Petersson, H. Nakatsuji, M. Caricato, X. Li, H. P. Hratchian, A. F. Izmaylov, J. Bloino, G. Zheng, J. L. Sonnenberg, M. Hada, M. Ehara, K. Toyota, R. Fukuda, J. Hasegawa, M. Ishida, T. Nakajima, Y. Honda, O. Kitao, H. Nakai, T. Vreven, J. A. Montgomery Jr, J. E. Peralta, F. Ogliaro, M. J. Bearpark, J. Heyd, E. N. Brothers, K. N. Kudin, V. N. Staroverov, R. Kobayashi, J. Normand, K. Raghavachari, A. P. Rendell, J. C. Burant, S. S. Iyengar, J. Tomasi, M. Cossi, N. Rega, N. J. Millam, M. Klene, J. E. Knox, J. B. Cross, V. Bakken, C. Adamo, J. Jaramillo, R. Gomperts, R. E. Stratmann, O. Yazyev, A. J. Austin, R. Cammi, C. Pomelli, J. W. Ochterski, R. L. Martin, K. Morokuma, V. G. Zakrzewski, G. A. Voth, P. Salvador, J. J. Dannenberg, S. Dapprich, A. D. Daniels, Ö. Farkas, J. B. Foresman, J. V. Ortiz, J. Cioslowski and D. J. Fox, *Gaussian 09, Revision B.01*, 2009, Gaussian, Inc., Wallingford CT, U.S.A.

2 S. Huang, Q. Zhang, Y. Shiota, T. Nakagawa, K. Kuwabara, K. Yoshizawa and C. Adachi, *J. Chem. Theory. Comput.*, 2013, **9**, 3872-3877.

3 Q. Zhang, B. Li, S. Huang, H. Nomura, H. Tanaka and C. Adachi, *Nat. Photon.*, 2014, **8**, 326-332.

4 T. Lu and F. Chen, *J. Comput. Chem.*, 2012, **33**, 580-592.

5 Becke, A. D. *Phys. Rev. A* 1988, **38**, 3098−3100.

6 Lee, C., Yang, W., Parr, R. G. *Phys. Rev. B*, 1988, **37**, 785−789.

7 Schultz, N., Zhao, Y., Truhlar, D. G. *J. Phys. Chem. A*, 2005, **109**, 11127−11143.

8 Staroverov, V. N., Scuseria, G. E., Tao, J., Perdew, J. P. *J. Chem. Phys.*, 2003, **119**, 12129−12137.

9 Reiher, M., Salomon, O., Hess, B. A. *Theor. Chem. Acc.*, 2001, **107**, 48−55.

10 Becke, A. D. *J. Chem. Phys*., 1993, **98**, 5648−5652.

11 Adamo, C., Scuseria, G. E. *J. Chem. Phys.*, 1999, **111**, 2889−2899.

12 Perdew, J. P., Burke, K., Ernzerhof, M. *Phys. Rev. Lett.*, 1996, **77**, 3865−3868.

13 Zhao, Y., Truhlar, D. G. *J. Phys. Chem. A*., 2004, **108**, 6908−6918.

14 Boese, A. D., Martin, J. M. L. *J. Chem. Phys*., 2004, **121**, 3405−3416.

15 Zhao, Y., Truhlar, D. G. *Theor. Chem. Acc.*, 2008, **120**, 215−241.

16 Zhao, Y., Truhlar, D. G. *J. Phys. Chem. A*., 2006, **110**, 13126−13130.

17 K. Masui, H. Nakanotani and C. Adachi, *Org. Electron.,* **2013***,* 14, 2721-2726.

18 Y. Zhang and S. R. Forrest, *Phys Rev Lett,* 2012, **108**, 267404.

19 D. Kasemann, R. Brückner, H. Fröb and K. Leo, *Phys. Rev. B.*, 2011, **84**.

20 C. M. Cardona, W. Li, A. E. Kaifer, D. Stockdale and G. C. Bazan, *Adv. Mater.*, 2011, **23**, 2367-2371.

21 A. P. Kulkarni, X. Kong and S. A. Jenekhe, *Adv. Funct. Mater.,* 2006, **16**, 1057-1066.

22 T. Higuchi, H. Nakanotani and C. Adachi, *Adv. Mater.*, 2015, **27**, 2019-2023.

23 J.-L. Brédas, *Mater. Horiz.*, 2014, **1***, 17-19.*

24 W. Li, Y. Pan, R. Xiao, Q. Peng, S. Zhang, D. Ma, F. Li, F. Shen, Y. Wang, B. Yang and Y. Ma, *Adv. Funct. Mater.,* 2014, **24,** 1609-1614.

4. Computation Geometry Data

Geometry Data for DC-TC in S_0

0 1

C	1.84236400	-1.16163200	-1.56465200
C	2.31287800	-1.49367200	-0.28268400
C	2.72232000	-0.53842500	-2.46757200
C	3.62125900	-1.20313000	0.08631700
H	1.65630900	-1.97843800	0.42957600
C	4.03300700	-0.26118300	-2.11041900
H	2.35570900	-0.29399000	-3.45923500
C	4.49588200	-0.58781700	-0.82316500
H	3.96786300	-1.43348000	1.08794200
H	4.71461100	0.18651400	-2.82573600
C	-1.90824900	-1.63882600	-0.89807200
C	-2.37418700	-0.43192000	-1.44729400
C	-2.77320400	-2.38835800	-0.08053400
C	-3.66800500	0.00761100	-1.19059900
H	-1.73680900	0.14747600	-2.10398700
C	-4.06161200	-1.95088700	0.18494600
H	-2.40219400	-3.31112700	0.35355000
C	-4.52392700	-0.74445100	-0.37072700
H	-4.03224800	0.92206900	-1.64631700
H	-4.71102900	-2.51995600	0.84168400
C	-7.43424500	3.38338400	1.16040300
C	-6.03809100	3.28782900	0.98983200
C	-5.40519000	2.11777200	0.56732300
C	-6.19644300	1.00303100	0.29698300
C	-7.59920200	1.05537000	0.47739700
C	-8.20242100	2.24024300	0.90167600
H	-5.41207300	4.14889700	1.19407800
H	-4.32578500	2.08777000	0.46593300
H	-9.28057900	2.26345900	1.03573600
C	-7.00476400	-1.06955900	-0.18255000
C	-7.18567900	-2.39197000	-0.58306500
C	-8.48441900	-2.90290000	-0.59888900
C	-9.61181700	-2.13766300	-0.23714900
C	-9.40160200	-0.80462800	0.14034900
C	-8.11499600	-0.26456100	0.16675900
H	-6.35120600	-3.01416200	-0.88780800
H	-8.61238100	-3.93330800	-0.90985500
H	-10.24362800	-0.17329300	0.41082300
C	8.85299900	-2.53119500	1.43273600
C	7.61966900	-3.16429000	1.17708600

C	6.54690300	-2.51439400	0.56517500
C	6.70866100	-1.17900900	0.20134600
C	7.94018800	-0.51739200	0.42048000
C	8.99415500	-1.19481900	1.03565900
H	7.48038900	-4.20193000	1.45740500
H	5.62308500	-3.04941800	0.37375900
H	9.93278000	-0.67192100	1.19790600
C	6.49201500	0.92224100	-0.64549100
C	6.03833100	2.12025000	-1.19419700
C	6.91915100	3.20231000	-1.23312500
C	8.23598600	3.13469000	-0.73477100
C	8.65746600	1.92403200	-0.16849700
C	7.80078700	0.82359400	-0.11561200
H	5.02834700	2.22612000	-1.57515000
H	6.55546800	4.12698200	-1.66632500
H	9.65930800	1.83224300	0.24247500
N	-5.83356100	-0.29495300	-0.10426000
N	5.82473500	-0.30023200	-0.45011300
C	-8.12573600	4.67983400	1.62622200
C	-7.12899600	5.83597300	1.83352300
C	-9.16032600	5.12584800	0.56567900
C	-8.84877500	4.42489100	2.97025200
H	-6.38763300	5.60212700	2.60600100
H	-6.59499500	6.08517300	0.90939500
H	-7.66767600	6.73418000	2.15580800
H	-9.93054200	4.36507700	0.40041000
H	-9.66484000	6.04566100	0.88651400
H	-8.67473400	5.32247600	-0.39702800
H	-9.35514000	5.33603900	3.31244200
H	-9.60407100	3.63667000	2.88381100
H	-8.13647100	4.12034600	3.74558100
C	-11.04286700	-2.71032500	-0.25218800
C	-11.64882700	-2.62251600	1.16878400
C	-11.91795800	-1.89294000	-1.23209300
C	-11.08169900	-4.18513300	-0.69629900
H	-11.05458900	-3.20224600	1.88431700
H	-11.69064400	-1.58970500	1.53070000
H	-12.67151800	-3.01943500	1.17462300
H	-11.51921100	-1.94723300	-2.25148700
H	-12.94344100	-2.28250300	-1.24599800
H	-11.96788200	-0.83593200	-0.95012500
H	-12.11681100	-4.54477600	-0.68941000
H	-10.69472300	-4.31641100	-1.71326800
H	-10.50439400	-4.82986600	-0.02380600

C	10.02869400	-3.25381200	2.11915400
C	11.26013800	-3.24928700	1.18229100
C	10.38550700	-2.52083000	3.43425900
C	9.69874700	-4.71908200	2.46340900
H	11.03993600	-3.76937500	0.24312700
H	11.57940700	-2.23185200	0.93302400
H	12.10727900	-3.75534200	1.66162700
H	9.53367900	-2.51747400	4.12393100
H	11.22667700	-3.01686500	3.93438700
H	10.67224400	-1.47925300	3.25443000
H	10.56382200	-5.18777600	2.94601700
H	8.85308200	-4.79676600	3.15626200
H	9.46292400	-5.30571400	1.56810800
C	9.20490500	4.33244600	-0.78487700
C	8.57528200	5.57156800	-1.44966900
C	9.62098500	4.72133200	0.65380700
C	10.46547700	3.94406900	-1.59385700
H	8.28207700	5.37438100	-2.48716800
H	7.69204600	5.92288400	-0.90421600
H	9.30186600	6.39184700	-1.46275900
H	10.11305900	3.89272100	1.17388100
H	10.32117200	5.56567400	0.63487000
H	8.74802700	5.01598600	1.24737000
H	11.16881900	4.78504600	-1.63419400
H	10.98995800	3.09296600	-1.14715700
H	10.20212800	3.67086500	-2.62205600
C	0.44623100	-1.39315100	-2.01210800
O	0.04696000	-1.05377700	-3.12009000
C	-0.52925500	-2.15313900	-1.09166700
O	-0.14296000	-3.20101700	-0.58665100

Geometry Data for DC-TC in S_1

0 1

C	-3.95869900	0.31827500	-0.88693800
C	-2.56569300	0.30246000	-0.79456300
C	-1.94442700	-0.08871900	0.40395200
C	-2.74395200	-0.43952700	1.51455300
C	-4.13166500	-0.41991600	1.42107100
C	-4.75191000	-0.04196200	0.21552300
H	-4.43716900	0.60325900	-1.82094600
H	-1.97252600	0.58811200	-1.65716200
H	-2.26137800	-0.72166900	2.44659700
H	-4.74399700	-0.67965800	2.28149700
C	-0.46317200	-0.12167900	0.60469000

O	0.00955500	-0.18533100	1.74502600
C	0.46317000	-0.12146700	-0.60476900
O	-0.00950200	-0.18484100	-1.74513900
C	1.94443300	-0.08863700	-0.40400200
C	2.56567200	0.30261100	0.79450500
C	2.74397200	-0.43948000	-1.51456900
C	3.95867500	0.31839700	0.88692000
H	1.97248800	0.58834400	1.65706100
C	4.13168900	-0.41986900	-1.42105600
H	2.26143600	-0.72162300	-2.44663200
C	4.75191000	-0.04189200	-0.21550800
H	4.43712400	0.60342000	1.82092700
H	4.74401900	-0.67961000	-2.28147900
N	-6.15757400	-0.02158700	0.11808600
C	-7.01605300	-1.05479700	0.50144300
C	-6.93034800	1.04051000	-0.35884400
C	-6.72415600	-2.33075500	0.99435400
C	-8.35773500	-0.65830200	0.26732100
C	-6.54201700	2.30647900	-0.80994300
C	-8.30285600	0.69021800	-0.28178100
C	-7.79511100	-3.18797900	1.27071900
H	-5.70017900	-2.66046700	1.15108700
C	-9.40795400	-1.53703100	0.55602700
C	-7.54583800	3.20031400	-1.19977000
H	-5.49717300	2.60334700	-0.84906800
C	-9.28390200	1.60458300	-0.68102100
C	-9.14542600	-2.81844400	1.06765600
H	-7.56062100	-4.17773100	1.65168500
H	-10.43296000	-1.21751700	0.37276300
C	-8.92185300	2.87722500	-1.15119800
H	-7.23899000	4.18240600	-1.54764600
H	-10.33363800	1.32131300	-0.61638300
C	-10.32078300	-3.76645200	1.38684800
C	-10.02250500	3.87150500	-1.57828400
C	-9.84174100	-5.14997300	1.87976300
C	-11.20177500	-3.13559300	2.49700800
C	-11.17968800	-3.97989900	0.11327200
C	-10.84957200	3.26094300	-2.73958500
C	-10.96045800	4.14841400	-0.37431000
C	-9.44161200	5.22021700	-2.05738300
H	-9.22398300	-5.65490000	1.12502800
H	-9.25971600	-5.06933800	2.80751800
H	-10.71289000	-5.78646400	2.08300600
H	-11.60150800	-2.16161600	2.18666900

H	-12.05078400	-3.79422700	2.72843400
H	-10.61832700	-2.98593400	3.41532000
H	-12.02237800	-4.65037100	0.33308700
H	-11.59059500	-3.03312300	-0.25940500
H	-10.57948300	-4.42941600	-0.68903600
H	-10.21097500	3.07137600	-3.61269800
H	-11.31366600	2.31086900	-2.44549500
H	-11.65045300	3.95150300	-3.03937700
H	-10.39815100	4.58222300	0.46330400
H	-11.75175500	4.85411900	-0.66371200
H	-11.44111600	3.22763300	-0.01982200
H	-10.26328200	5.88762300	-2.34906600
H	-8.86872700	5.71924100	-1.26445600
H	-8.78808100	5.09094400	-2.93041600
N	6.15757500	-0.02154800	-0.11802700
C	6.93038200	1.04054100	0.35886600
C	7.01602500	-1.05477100	-0.50140500
C	6.54209900	2.30652900	0.80995800
C	8.30288100	0.69019900	0.28181600
C	6.72407400	-2.33070600	-0.99433800
C	8.35771800	-0.65832400	-0.26728500
C	7.54595400	3.20032600	1.19978600
H	5.49727200	2.60345000	0.84908500
C	9.28396000	1.60452900	0.68105800
C	7.79499500	-3.18796700	-1.27072100
H	5.70007100	-2.66033500	-1.15109600
C	9.40790300	-1.53709400	-0.55599400
C	8.92195700	2.87718800	1.15122600
H	7.23913700	4.18242900	1.54765900
H	10.33368400	1.32121900	0.61641700
C	9.14532600	-2.81849300	-1.06762900
H	7.56046700	-4.17770100	-1.65170800
H	10.43292200	-1.21761400	-0.37274400
C	10.02263600	3.87144100	1.57830600
C	10.32064400	-3.76653900	-1.38685900
C	9.44176600	5.22016800	2.05738700
C	10.84968000	3.26087300	2.73962100
C	10.96060100	4.14831900	0.37433500
C	11.20193300	-3.13543700	-2.49664600
C	11.17927000	-3.98048900	-0.11317800
C	9.84153900	-5.14985200	-1.88029700
H	8.86888300	5.71918800	1.26445600
H	8.78823800	5.09091800	2.93042700
H	10.26344800	5.88756600	2.34905500

H	11.31373700	2.31077400	2.44555500
H	11.65058900	3.95141000	3.03939400
H	10.21107800	3.07135400	3.61274000
H	11.75192000	4.85399700	0.66374200
H	11.44123000	3.22752300	0.01984600
H	10.39831100	4.58214900	-0.46327900
H	10.61867700	-2.98538700	-3.41501700
H	11.60174200	-2.16162000	-2.18590100
H	12.05089200	-3.79411400	-2.72813500
H	10.57882500	-4.43009700	0.68890000
H	12.02186600	-4.65107400	-0.33301300
H	11.59030400	-3.03389800	0.25982600
H	10.71265400	-5.78640600	-2.08348300
H	9.22350000	-5.65490400	-1.12587600
H	9.25978100	-5.06887000	-2.80819000

Geometry Data for DC-ACR in S_0

0 1

C	-3.66640400	-0.29903700	-0.85194700
C	-2.35122700	-0.75072900	-0.76744000
C	-1.88456500	-1.34193200	0.41828600
C	-2.75687000	-1.46861600	1.51336800
C	-4.06964600	-1.02284100	1.42490500
C	-4.53078200	-0.43531900	0.23911600
H	-4.03621600	0.15651900	-1.76525800
H	-1.69740800	-0.65860500	-1.62610600
H	-2.38170700	-1.92319600	2.42436700
H	-4.74978300	-1.12253700	2.26529500
C	-0.48455800	-1.81524300	0.59994900
O	-0.08244800	-2.27870800	1.65860400
C	0.48456800	-1.81532800	-0.60006900
O	0.08258100	-2.27893500	-1.65870000
C	1.88457300	-1.34195500	-0.41833300
C	2.35098300	-0.75010600	0.76716200
C	2.75711100	-1.46923400	-1.51315800
C	3.66614600	-0.29837200	0.85171000
H	1.69699900	-0.65752900	1.62565400
C	4.06988100	-1.02344000	-1.42464700
H	2.38214100	-1.92429000	-2.42399900
C	4.53076400	-0.43527400	-0.23908000
H	4.03575600	0.15765800	1.76486500
H	4.75019900	-1.12363500	-2.26483200
C	-6.52252300	-2.18037900	-0.68936000
C	-6.87496400	-0.86450500	-0.32930700

C	-7.47739200	-3.07303900	-1.16117400
C	-8.21664900	-0.44673900	-0.44530300
C	-8.80660200	-2.67334300	-1.28495700
H	-7.17467700	-4.08117100	-1.43117800
C	-6.19536400	1.34021200	0.52980100
C	-9.15137900	-1.37312200	-0.92630100
H	-9.56378400	-3.35908400	-1.65350800
C	-5.17621000	2.18888900	1.00602000
C	-7.51817000	1.82185900	0.44477900
H	-10.18836400	-1.06485000	-1.02363800
C	-5.45486200	3.49344200	1.39564600
H	-4.15893900	1.82294900	1.07191900
C	-7.76154400	3.14186200	0.84616300
C	-6.75746200	3.98255000	1.31877500
H	-4.64749900	4.12353700	1.75922700
H	-8.77557600	3.52611700	0.78527700
H	-6.99068800	4.99961400	1.61971000
H	-5.49358700	-2.50611400	-0.59914100
N	-5.88587500	0.02149800	0.14324400
C	5.17628000	2.18881700	-1.00628200
C	6.19538400	1.34020400	-0.52985200
C	5.45496300	3.49334300	-1.39598000
C	7.51819200	1.82184300	-0.44480000
C	6.75754700	3.98247600	-1.31899000
H	4.64763700	4.12339300	-1.75972200
C	6.87492500	-0.86446600	0.32942100
C	7.76159500	3.14182400	-0.84623700
H	6.99078800	4.99953100	-1.61994600
C	6.52241100	-2.18026400	0.68968600
C	8.21664000	-0.44676300	0.44530200
H	8.77561900	3.52608800	-0.78527500
C	7.47726700	-3.07295700	1.16146300
H	5.49342400	-2.50589300	0.59966500
C	9.15135900	-1.37318400	0.92624700
C	8.80652600	-2.67335800	1.28502100
H	7.17450600	-4.08103100	1.43163200
H	10.18837700	-1.06498400	1.02347700
H	9.56370500	-3.35913400	1.65351700
H	4.15902500	1.82285000	-1.07229600
N	5.88585700	0.02154800	-0.14312700
C	8.68223300	0.96601500	0.07073800
C	-8.68223800	0.96602400	-0.07068700
C	9.76682400	0.86341300	-1.03623200
H	10.62439700	0.27453100	-0.69452900

H	9.36052500	0.38275000	-1.93211400
H	10.13751000	1.85350200	-1.32094300
C	9.29238300	1.64687800	1.32633300
H	9.65536700	2.65339600	1.09301500
H	8.54485300	1.73158700	2.12181300
H	10.13781800	1.06993400	1.71524700
C	-9.76675000	0.86336100	1.03635500
H	-9.36038700	0.38267300	1.93219500
H	-10.13743800	1.85343400	1.32112500
H	-10.62433000	0.27447100	0.69467800
C	-9.29250800	1.64691600	-1.32621100
H	-8.54504300	1.73169700	-2.12174300
H	-10.13795000	1.06995700	-1.71508200
H	-9.65552100	2.65340400	-1.09281400

Geometry Data for DC-ACR in S_1

0 1

C	4.01332700	-0.16132500	-1.00705400
C	2.61946000	-0.15862300	-0.91738000
C	1.97012000	-0.00891100	0.33139100
C	2.78401600	0.13501300	1.48335300
C	4.17226000	0.14116800	1.40593300
C	4.78194400	-0.00818200	0.15203800
H	4.50349300	-0.27885900	-1.97239700
H	2.02202000	-0.27089400	-1.81310100
H	2.28078700	0.24313000	2.44015000
H	4.78362500	0.25904300	2.29923300
C	0.48531200	0.00530500	0.58191000
O	0.10127800	0.08460200	1.77306800
C	-0.45495500	-0.05159400	-0.56100500
O	-0.06546500	-0.09353200	-1.74430100
C	-1.95444000	-0.03730800	-0.32037900
C	-2.59715800	-0.15049500	0.92990100
C	-2.75949000	0.08036000	-1.47360800
C	-3.99466200	-0.14508400	1.01317900
H	-1.99637300	-0.24003500	1.82602100
C	-4.15145700	0.09652100	-1.39006000
H	-2.25757100	0.15632600	-2.43450000
C	-4.77636100	-0.01779800	-0.14014500
H	-4.48829600	-0.23648500	1.97946200
H	-4.76453900	0.19430400	-2.28444600
C	6.10340000	2.39543600	-0.28853600
C	6.87329000	1.20324400	-0.15279500
C	6.73297900	3.60974600	-0.50194700

C	8.29614600	1.25688400	-0.23382800
C	8.13447700	3.67165900	-0.58252800
H	6.13420000	4.51056500	-0.60677800
C	6.90364300	-1.20233900	0.18302900
C	8.89129600	2.50385900	-0.44698600
H	8.63473200	4.62259600	-0.74959600
C	6.16210900	-2.40428600	0.37748000
C	8.32774800	-1.24048900	0.12174200
H	9.97471200	2.56882700	-0.50627200
C	6.82140500	-3.61436800	0.50907200
H	5.08066300	-2.36885000	0.41843100
C	8.95358600	-2.48273400	0.26407200
C	8.22488400	-3.66073100	0.45468300
H	6.24450200	-4.52380000	0.65474400
H	10.03881200	-2.53544800	0.22691600
H	8.74881400	-4.60777700	0.55971800
H	5.02333500	2.35136300	-0.22827300
N	6.22491700	-0.00234400	0.05607200
C	-6.19662700	-2.42337300	-0.34329700
C	-6.90892000	-1.21288000	-0.17157300
C	-6.86743600	-3.63745300	-0.47340400
C	-8.32270700	-1.24182800	-0.12619100
C	-8.26510400	-3.67902200	-0.43630700
H	-6.28844500	-4.55013200	-0.60417500
C	-6.87118000	1.21149700	0.14617900
C	-8.96517300	-2.48349900	-0.26342100
H	-8.80190700	-4.61976300	-0.53902900
C	-6.12258300	2.40437100	0.28180000
C	-8.28336300	1.27015200	0.20961700
H	-10.05316900	-2.51149200	-0.23558300
C	-6.75566000	3.63003600	0.47719100
H	-5.03954000	2.36256600	0.23546600
C	-8.88728000	2.52295200	0.40573800
C	-8.15107900	3.70141800	0.54131300
H	-6.14910800	4.52823300	0.57965300
H	-9.97385100	2.57390400	0.44958400
H	-8.65845400	4.65186000	0.69273300
H	-5.11227500	-2.40244300	-0.37246200
N	-6.20854800	-0.00683000	-0.04777900
C	-9.17195200	0.02511100	0.06221300
C	9.16920200	0.01535800	-0.08217100
C	-10.10005600	0.20653200	-1.17425900
H	-10.73384600	1.09497200	-1.05802000
H	-9.50263000	0.32501800	-2.08664700

H	-10.75574600	-0.66442300	-1.30082000
C	-10.04863500	-0.13313700	1.33882500
H	-10.70740200	-1.00653300	1.25380500
H	-9.41508600	-0.26297600	2.22486700
H	-10.67955200	0.75160000	1.49133200
C	10.10212800	0.20504000	1.15548400
H	9.51135900	0.33355100	2.07009500
H	10.75274000	-0.66825000	1.27942300
H	10.73621900	1.08897800	1.02154500
C	10.03948400	-0.15806600	-1.36595300
H	9.40531100	-0.29141500	-2.25019400
H	10.67344800	0.72240500	-1.51991900
H	10.69230200	-1.03282600	-1.26904500

Geometry Data for PyCN-TC in S_0

0 1

C	-0.13996900	4.70681200	-0.35053700
C	1.25393000	4.54690600	-0.04279200
N	2.00640700	5.63086200	0.18248000
C	1.48869800	6.84354200	-0.02908800
C	0.19151700	6.98113700	-0.56510800
N	-0.60812100	5.91695700	-0.67715800
C	-0.32796700	8.25499700	-0.98220700
N	-0.73934900	9.28784800	-1.32088600
C	2.30794200	7.98342000	0.28181600
N	2.96316000	8.90947400	0.53432900
C	-1.15282200	3.62702600	-0.29783300
C	-1.11518500	2.62399800	0.68584100
C	-2.23360700	3.64273000	-1.19576100
C	-2.10864600	1.65482300	0.75467900
H	-0.30343200	2.59858200	1.40518400
C	-3.22718100	2.67294400	-1.13459900
H	-2.29427900	4.43495500	-1.93430300
C	-3.17262800	1.66354200	-0.16110700
H	-2.05754500	0.87605600	1.50804200
H	-4.06332700	2.70493000	-1.82497700
C	1.97162800	3.25401200	0.00936300
C	1.65988800	2.19466400	-0.86055600
C	3.05492800	3.09609500	0.89132600
C	2.39720800	1.01767700	-0.84443200
H	0.85610500	2.30229300	-1.58091400
C	3.77487900	1.90885200	0.93428300
H	3.31555400	3.91173100	1.55726600
C	3.45564500	0.85430600	0.06391500

H	2.17376600	0.22720800	-1.55268400
H	4.57726700	1.78499700	1.65354400
C	3.08361700	-4.40580700	0.21967900
C	2.05957000	-3.43748500	0.20263400
C	2.31575200	-2.06609800	0.15613300
C	3.64386800	-1.64616700	0.11694400
C	4.69884100	-2.58895400	0.15381500
C	4.40987800	-3.95350700	0.20087800
H	1.02268400	-3.75223100	0.22839400
H	1.49382800	-1.35820400	0.15940500
H	5.22995900	-4.66597100	0.22841800
C	5.58969000	-0.46520900	0.11609200
C	6.57571000	0.51738500	0.05417800
C	7.91084200	0.11089000	0.05653900
C	8.29763800	-1.24369500	0.11058600
C	7.28386400	-2.21062300	0.15052900
C	5.93929200	-1.83608500	0.14937100
H	6.32738900	1.57146700	-0.00669700
H	8.66879900	0.88431800	0.01039600
H	7.53619700	-3.26736000	0.17657800
C	-6.54181900	-0.88536100	3.00262500
C	-5.64060700	0.15439500	3.30928600
C	-4.80455400	0.73704700	2.35555500
C	-4.86565900	0.25843000	1.04828500
C	-5.77328100	-0.76968500	0.69830100
C	-6.59584300	-1.33219800	1.67552800
H	-5.58325000	0.53318800	4.32316900
H	-4.13874300	1.54662900	2.63517800
H	-7.28715100	-2.12146100	1.39281700
C	-4.63763600	-0.09409000	-1.18795500
C	-4.23724100	-0.10075400	-2.52252900
C	-4.85450400	-1.00260900	-3.39053900
C	-5.85440300	-1.90405800	-2.97209400
C	-6.22475700	-1.88621500	-1.62064400
C	-5.62516500	-0.99727100	-0.72706700
H	-3.46315000	0.56541500	-2.88832200
H	-4.53674700	-0.99617900	-4.42676300
H	-6.98289800	-2.57227000	-1.25280600
N	4.18805400	-0.34954600	0.09601600
N	-4.17385000	0.67093000	-0.10367200
C	2.79449500	-5.91915600	0.26284200
C	1.28563500	-6.23056300	0.28439500
C	3.40521500	-6.59594500	-0.98753500
C	3.42727700	-6.53121000	1.53540700

H	0.79245800	-5.80473900	1.16570700
H	0.77689800	-5.85181100	-0.60946300
H	1.13387700	-7.31537900	0.31479100
H	4.48874000	-6.44702200	-1.04302600
H	3.21768100	-7.67664900	-0.96877400
H	2.96584700	-6.19040400	-1.90607900
H	3.23878600	-7.61111200	1.57494200
H	4.51174900	-6.38191000	1.56435900
H	3.00442400	-6.07873100	2.43963500
C	9.77515700	-1.68324700	0.11774200
C	10.06836200	-2.48757900	1.40662200
C	10.05598100	-2.57464400	-1.11545800
C	10.74453900	-0.48691900	0.07094400
H	9.89226800	-1.87564900	2.29869100
H	9.43607400	-3.37830200	1.48343100
H	11.11410500	-2.81885000	1.42214800
H	9.86586400	-2.02732300	-2.04574900
H	11.10308200	-2.90194400	-1.12101900
H	9.42780800	-3.47163300	-1.12257100
H	11.77841800	-0.84977900	0.08098600
H	10.61323700	0.10907600	-0.83929800
H	10.61873500	0.17470500	0.93557800
C	-7.45635200	-1.53029400	4.06282100
C	-8.93671900	-1.34838500	3.65090800
C	-7.13742700	-3.04088700	4.16717300
C	-7.27067600	-0.90604200	5.45902200
H	-9.19446500	-0.28599100	3.57405800
H	-9.15145900	-1.81394200	2.68321900
H	-9.59988600	-1.80680900	4.39490600
H	-6.09652300	-3.20115000	4.47085600
H	-7.78646900	-3.51965500	4.91084400
H	-7.28829300	-3.55615800	3.21261200
H	-7.93827600	-1.39897900	6.17463000
H	-6.24576500	-1.02676000	5.82793900
H	-7.51364100	0.16267000	5.46420800
C	-6.53794100	-2.89094900	-3.93899800
C	-5.98213200	-2.79055900	-5.37235800
C	-6.31947900	-4.34037800	-3.44331300
C	-8.05527300	-2.59254100	-3.99080100
H	-6.13701700	-1.79460200	-5.80261800
H	-4.91064000	-3.01817500	-5.41162100
H	-6.49608300	-3.51135800	-6.01818200
H	-6.73758100	-4.49845700	-2.44352800
H	-6.80381900	-5.05321600	-4.12203600

H	-5.25165000	-4.58337300	-3.40026500
H	-8.56114700	-3.29242300	-4.66743100
H	-8.52251100	-2.68582000	-3.00462800
H	-8.24201500	-1.57488700	-4.35241600

Geometry Data for PyCN-TC in S_1

0 1

C	0.18193100	4.61496700	0.25490700
C	-1.20745000	4.44183500	-0.21601400
N	-1.89280200	5.53535500	-0.58781300
C	-1.41017300	6.73260700	-0.32221800
C	-0.17511500	6.88284900	0.42641900
N	0.59931500	5.81569400	0.63277300
C	0.28227500	8.15946300	0.88308200
N	0.63289000	9.20204400	1.26026600
C	-2.15885800	7.87588900	-0.77614100
N	-2.75279800	8.80309900	-1.14289700
C	1.20862000	3.56045300	0.22945500
C	1.16195000	2.49700500	-0.70811900
C	2.31907700	3.62389900	1.10948700
C	2.16570000	1.53472700	-0.75353700
H	0.34083600	2.44246300	-1.41729600
C	3.31332900	2.65749000	1.08073900
H	2.38642100	4.45643100	1.80448200
C	3.24525100	1.59633100	0.14815700
H	2.10872200	0.71711000	-1.46834100
H	4.16777600	2.72726700	1.74992100
C	-1.95528000	3.18578200	-0.21548400
C	-1.59788300	2.07936500	0.60650200
C	-3.13343000	3.06123500	-1.00642000
C	-2.35889900	0.91783900	0.62490400
H	-0.73792600	2.15223400	1.26541900
C	-3.88987100	1.90170400	-0.99896200
H	-3.42297000	3.89593000	-1.63815000
C	-3.51041700	0.81039600	-0.18046300
H	-2.09527200	0.10189100	1.29438100
H	-4.75676600	1.80896800	-1.64928000
C	-3.29679400	-4.43188000	-0.45494300
C	-2.25135900	-3.48645000	-0.54803700
C	-2.46950900	-2.10657400	-0.46259600
C	-3.78328900	-1.66418900	-0.26285700
C	-4.86170300	-2.58741800	-0.19230700
C	-4.61623200	-3.95372000	-0.28469800
H	-1.23139900	-3.82726600	-0.69641500

H	-1.64151300	-1.41011800	-0.55937500
H	-5.44955900	-4.65194700	-0.22954400
C	-5.66497000	-0.42949200	-0.02647100
C	-6.59301400	0.60126200	0.16860400
C	-7.94128400	0.24888800	0.30074300
C	-8.38800900	-1.09067600	0.26070300
C	-7.42345500	-2.11099500	0.09570600
C	-6.07727700	-1.78920800	-0.04280900
H	-6.28548100	1.64121800	0.22999500
H	-8.65524100	1.05313000	0.44816300
H	-7.72979800	-3.15537000	0.08451700
C	6.53382800	-1.08742400	-2.96151300
C	5.59367900	-0.08900900	-3.30263600
C	4.78003900	0.54034800	-2.35395100
C	4.90664300	0.14420400	-1.01742500
C	5.86017600	-0.83715100	-0.63691900
C	6.65897600	-1.44643300	-1.60415500
H	5.49230000	0.21965300	-4.33871200
H	4.08765100	1.32196500	-2.65403900
H	7.38740600	-2.19774400	-1.30261100
C	4.77150000	-0.04354600	1.23585000
C	4.39885700	0.04428500	2.58215700
C	5.07174900	-0.76802900	3.50147900
C	6.09154000	-1.66972400	3.12146900
C	6.42657900	-1.75621400	1.75487000
C	5.77257300	-0.95834600	0.81627300
H	3.60682500	0.71155900	2.91108400
H	4.78233700	-0.69431400	4.54528900
H	7.19517500	-2.45297100	1.42369500
N	-4.27986500	-0.36687800	-0.16346400
N	4.25363000	0.61761900	0.12143800
C	-3.04954700	-5.95031700	-0.53538100
C	-1.55656800	-6.29421900	-0.73247500
C	-3.53124300	-6.61516700	0.78178000
C	-3.84538200	-6.54166300	-1.72913200
H	-1.16396800	-5.87204600	-1.66713900
H	-0.94313900	-5.92918600	0.10186000
H	-1.44065500	-7.38453400	-0.78135000
H	-4.60135900	-6.44369300	0.95463200
H	-3.36555800	-7.70033500	0.73590900
H	-2.97819400	-6.21746800	1.64293800
H	-3.68406400	-7.62709200	-1.78272700
H	-4.92360400	-6.36569400	-1.62634400
H	-3.51580100	-6.09553500	-2.67677200

C	-9.87283400	-1.47764700	0.39903800
C	-10.32167400	-2.23808300	-0.87747100
C	-10.05111500	-2.40038900	1.63396300
C	-10.78990700	-0.24743900	0.57665600
H	-10.21400300	-1.60304700	-1.76662600
H	-9.72940000	-3.14849000	-1.03480200
H	-11.37643500	-2.53139700	-0.78659700
H	-9.74943400	-1.88273800	2.55406100
H	-11.10510900	-2.69459200	1.73076900
H	-9.45165900	-3.31532000	1.54495600
H	-11.83174100	-0.58223200	0.66175200
H	-10.54371700	0.31532400	1.48689300
H	-10.72618500	0.43256000	-0.28321100
C	7.42734500	-1.77661400	-4.01172100
C	8.91738300	-1.49750000	-3.68146100
C	7.17535600	-3.30692100	-3.97674100
C	7.14702300	-1.27126400	-5.44412200
H	9.12647800	-0.41978700	-3.70663300
H	9.18707300	-1.87325300	-2.68628900
H	9.56430800	-1.99325700	-4.41839000
H	6.12752700	-3.53569100	-4.21274800
H	7.81393300	-3.80952300	-4.71625800
H	7.40385900	-3.72902400	-2.98992800
H	7.80386200	-1.79629600	-6.14979200
H	6.10864400	-1.46251500	-5.74613600
H	7.34632200	-0.19558500	-5.53912300
C	6.83867100	-2.55164300	4.14160100
C	6.30930200	-2.36225900	5.58033300
C	6.67218400	-4.04543500	3.75775000
C	8.34605700	-2.18411900	4.12521600
H	6.43644700	-1.32808400	5.92652100
H	5.24653900	-2.62845400	5.65736200
H	6.86870300	-3.01502100	6.26293600
H	7.08026100	-4.25716100	2.76146500
H	7.20312600	-4.67920400	4.48140100
H	5.61223700	-4.33253400	3.75994100
H	8.89440800	-2.81501500	4.83848500
H	8.78541100	-2.33317200	3.13061700
H	8.49246400	-1.13353500	4.40907900

Geometry Data for PyCN-ACR in S_0

0 1

C	0.69393700	3.64903900	0.17916100
C	-0.69407300	3.64902200	-0.17913300

N	-1.30119900	4.80552400	-0.46930100
C	-0.64486200	5.95384500	-0.28532500
C	0.64465700	5.95385600	0.28542000
N	1.30102800	4.80555100	0.46936400
C	1.30829900	7.17171000	0.66444600
N	1.83596000	8.16055200	0.97125800
C	-1.30855300	7.17169000	-0.66429200
N	-1.83625400	8.16052700	-0.97105500
C	1.56677700	2.44954200	0.21793800
C	1.44595800	1.42112900	-0.73164100
C	2.59815600	2.37752400	1.16915900
C	2.32771300	0.34408300	-0.72163000
H	0.67384800	1.47014400	-1.49231500
C	3.46857000	1.29143200	1.18773200
H	2.71147200	3.18161800	1.88834500
C	3.33994900	0.26869500	0.24168200
H	2.24358500	-0.44381800	-1.46358700
H	4.25821500	1.23074900	1.93027000
C	-1.56687600	2.44949900	-0.21794900
C	-1.44601400	1.42105100	0.73158600
C	-2.59826500	2.37749000	-1.16915900
C	-2.32774200	0.34398300	0.72154900
H	-0.67389900	1.47006000	1.49225500
C	-3.46864800	1.29137200	-1.18776400
H	-2.71161100	3.18160600	-1.88831400
C	-3.33998900	0.26860400	-0.24175400
H	-2.24358400	-0.44394200	1.46347700
H	-4.25829800	1.23069500	-1.93029600
C	5.73213700	0.40512800	-1.22286700
C	5.43909100	-0.76224900	-0.49045500
C	6.90369100	0.51040700	-1.96313900
C	6.34677300	-1.84090400	-0.50808100
C	7.80997700	-0.54763900	-1.99054500
H	7.10218800	1.42416200	-2.51678800
C	3.91658300	-1.98614800	1.00901500
C	7.51684700	-1.69962500	-1.26598300
H	8.72930400	-0.48045600	-2.56468500
C	2.71689900	-2.01974200	1.74715700
C	4.78041700	-3.10054600	1.03603900
H	8.22482900	-2.52307800	-1.29071700
C	2.37075500	-3.13429400	2.50176200
H	2.05107800	-1.16559600	1.72923300
C	4.39892700	-4.20619900	1.80738100
C	3.21434400	-4.24265300	2.53778100

H	1.43847900	-3.13025000	3.06024500
H	5.05356600	-5.07245200	1.83653700
H	2.95751500	-5.12110000	3.12227000
C	-2.71667500	-2.01995400	-1.74693200
C	-3.91642300	-1.98635600	-1.00889400
C	-2.37035700	-3.13460200	-2.50131700
C	-4.78016400	-3.10082800	-1.03583200
C	-3.21383700	-4.24304800	-2.53722400
H	-1.43803400	-3.13056000	-3.05972200
C	-5.43919800	-0.76233100	0.49020400
C	-4.39849700	-4.20657600	-1.80695000
H	-2.95686800	-5.12157100	-3.12153700
C	-5.73239000	0.40511800	1.22244200
C	-6.34682100	-1.84103500	0.50786100
H	-5.05305600	-5.07289200	-1.83603000
C	-6.90405000	0.51043700	1.96254000
H	-5.03883400	1.23685800	1.21037800
C	-7.51700700	-1.69971300	1.26558100
C	-7.81029500	-0.54764500	1.98995000
H	-7.10266000	1.42424900	2.51605500
H	-8.22494900	-2.52320100	1.29033600
H	-8.72970500	-0.48043000	2.56395200
N	4.24302100	-0.84315600	0.25216600
N	-4.24303400	-0.84326900	-0.25226500
H	5.03855000	1.23684200	-1.21079900
H	-2.05094500	-1.16573500	-1.72910400
C	6.10089100	-3.14706500	0.25739100
C	7.26981300	-3.37925100	1.25299700
C	6.04731200	-4.32324200	-0.75586400
H	7.32894100	-2.56068200	1.97762400
H	7.13733600	-4.31418900	1.80710600
H	8.22930000	-3.43849600	0.72901100
H	5.23042400	-4.18095900	-1.47094100
H	6.98240900	-4.40051800	-1.32027200
H	5.88769700	-5.27810600	-0.24411500
C	-6.10073400	-3.14731400	-0.25734200
C	-6.04719700	-4.32330000	0.75613600
C	-7.26950500	-3.37977700	-1.25306100
H	-5.23047500	-4.18078500	1.47135600
H	-6.98239900	-4.40060200	1.32036700
H	-5.88734700	-5.27822900	0.24458100
H	-7.32860800	-2.56133300	-1.97783100
H	-7.13687200	-4.31479500	-1.80699500
H	-8.22905700	-3.43902300	-0.72919400

Geometry Data for PyCN-ACR in S_1

0 1

C	-0.71588800	3.65726700	-0.25319800
C	0.68842900	3.63893100	0.17120400
N	1.23960800	4.82053300	0.56900300
C	0.59804500	5.93864200	0.32738000
C	-0.66640700	5.96270100	-0.34903200
N	-1.31975800	4.78716900	-0.54134400
C	-1.31212800	7.17626300	-0.72448900
N	-1.82307500	8.17260600	-1.04344300
C	1.22464100	7.16592500	0.78208000
N	1.72753000	8.14663600	1.13916200
C	-1.60548500	2.45342300	-0.27200100
C	-1.50637400	1.44766200	0.70877700
C	-2.62242000	2.35129000	-1.24034200
C	-2.38531700	0.36177600	0.70800400
H	-0.75325800	1.52181600	1.48926900
C	-3.50198200	1.26604700	-1.24278200
H	-2.72032700	3.14157600	-1.98053500
C	-3.38164600	0.26189600	-0.27182700
H	-2.31374400	-0.41006300	1.47210400
H	-4.28380300	1.18734200	-1.99594000
C	1.57514900	2.48825400	0.17906900
C	1.33441800	1.31111000	-0.59054400
C	2.78852500	2.53362500	0.93096500
C	2.22069500	0.24207300	-0.58045000
H	0.45303900	1.24737900	-1.22037500
C	3.67053600	1.46475700	0.95616700
H	3.00828700	3.43109300	1.50133000
C	3.38530000	0.30992900	0.20263300
H	2.02206300	-0.64241800	-1.18333800
H	4.57803400	1.51171300	1.55583100
N	4.28821700	-0.81353900	0.24228600
C	5.34972100	-0.85112000	-0.64797400
C	4.06018400	-1.81658700	1.17222400
C	5.52583700	0.22471800	-1.56635800
C	6.25890200	-1.95016800	-0.64663500
C	2.95798600	-1.69781600	2.06849200
C	4.91487400	-2.95579700	1.23425800
C	6.58279400	0.20967800	-2.46043500
H	4.83257300	1.05673200	-1.56183800
C	7.31037900	-1.92659100	-1.56754500
C	6.10951200	-3.13201400	0.30435900

C	2.70916300	-2.69084900	3.00039300
H	2.31515400	-0.82790400	2.01902100
C	4.62649700	-3.93641100	2.18850900
C	7.48356900	-0.86907800	-2.46568100
H	6.71001300	1.03748100	-3.15296900
H	8.01497300	-2.75410100	-1.58900500
C	7.40539800	-3.26610300	1.16396200
C	5.90910400	-4.43280100	-0.53607400
C	3.54333600	-3.82007400	3.06450100
H	1.86510800	-2.59000700	3.67751100
H	5.26169500	-4.81607000	2.25277500
H	8.31617700	-0.88607200	-3.16481600
H	7.56285300	-2.36900800	1.77405100
H	7.33148500	-4.13352700	1.82939700
H	8.27922900	-3.40839800	0.51861800
H	5.00783300	-4.35971400	-1.15603200
H	6.77247400	-4.59926300	-1.19026600
H	5.80977900	-5.30090800	0.12544600
H	3.35028800	-4.60521600	3.79148100
N	-4.27023700	-0.86137400	-0.27801700
C	-3.96472700	-1.96720300	-1.08241100
C	-5.41143400	-0.83581700	0.53834700
C	-2.80347500	-1.94979300	-1.89052300
C	-4.80213800	-3.10735300	-1.09809400
C	-5.69123500	0.31139000	1.31611700
C	-6.28919900	-1.94273000	0.59127000
C	-2.48039000	-3.03321600	-2.70540600
H	-2.16124500	-1.07522600	-1.87708600
C	-4.44495500	-4.18135700	-1.93033900
C	-6.07185800	-3.21626500	-0.24013400
C	-6.81317100	0.36066500	2.14142500
H	-5.02358400	1.16558200	1.26533300
C	-7.40870400	-1.85912700	1.43568400
C	-3.30300100	-4.16408300	-2.73380000
H	-1.58340400	-2.98537400	-3.32061300
H	-5.08568700	-5.06122200	-1.94780000
C	-5.93497700	-4.43596300	0.71724300
C	-7.30167600	-3.43277500	-1.16966800
C	-7.68417100	-0.73149100	2.21141100
H	-7.00071900	1.25790200	2.72846700
H	-8.08680300	-2.70931400	1.48528300
H	-3.06439000	-5.01511500	-3.36792500
H	-5.06830300	-4.30979800	1.37804200
H	-6.83183300	-4.54501400	1.33987100

H	-5.80392200	-5.36458900	0.14738300
H	-7.42914200	-2.57599600	-1.84272400
H	-7.17655100	-4.33653600	-1.77916400
H	-8.21942600	-3.54808000	-0.57953000
H	-8.56232200	-0.70655700	2.85282800
